# Pluripotent stem cell model of early hematopoiesis in Down syndrome reveals quantitative effects of short-form GATA1 protein on lineage specification

**DOI:** 10.1371/journal.pone.0247595

**Published:** 2021-03-29

**Authors:** Shiori Matsuo, Yoko Nishinaka-Arai, Yasuhiro Kazuki, Mitsuo Oshimura, Tatsutoshi Nakahata, Akira Niwa, Megumu K. Saito

**Affiliations:** 1 Department of Clinical Application, Center for iPS Cell Research and Application, Kyoto University, Kyoto, Japan; 2 Department of Human Health Sciences, Graduate School of Medicine, Kyoto University, Kyoto, Japan; 3 Chromosome Engineering Research Center, Tottori University, Tottori, Japan; 4 Division of Genome and Cellular Functions, Department of Molecular and Cellular Biology, School of Life Science, Faculty of Medicine, Tottori University, Tottori, Japan; 5 Drug Discovery Technology Development Office, Center for iPS Cell Research and Application, Kyoto University, Kyoto, Japan; University of Tennessee Health Science Center College of Medicine Memphis, UNITED STATES

## Abstract

Children with Down syndrome (DS) are susceptible to two blood disorders, transient abnormal myelopoiesis (TAM) and Down syndrome-associated acute megakaryocytic leukemia (DS-AMKL). Mutations in *GATA binding protein 1* (*GATA1*) have been identified as the cause of these diseases, and the expression levels of the resulting protein, short-form GATA1 (GATA1s), are known to correlate with the severity of TAM. On the other hand, despite the presence of *GATA1* mutations in almost all cases of DS-AMKL, the incidence of DS-AMKL in TAM patients is inversely correlated with the expression of GATA1s. This discovery has required the need to clarify the role of GATA1s in generating the cells of origin linked to the risk of both diseases. Focusing on this point, we examined the characteristics of *GATA1* mutant trisomy-21 pluripotent stem cells transfected with a doxycycline (Dox)-inducible GATA1s expression cassette in a stepwise hematopoietic differentiation protocol. We found that higher GATA1s expression significantly reduced commitment into the megakaryocytic lineage at the early hematopoietic progenitor cell (HPC) stage, but once committed, the effect was reversed in progenitor cells and acted to maintain the progenitors. These differentiation stage-dependent reversal effects were in contrast to the results of myeloid lineage, where GATA1s simply sustained and increased the number of immature myeloid cells. These results suggest that although *GATA1* mutant cells cause the increase in myeloid and megakaryocytic progenitors regardless of the intensity of GATA1s expression, the pathways vary with the expression level. This study provides experimental support for the paradoxical clinical features of *GATA1* mutations in the two diseases.

## Introduction

Children with Down syndrome (DS) are known to be susceptible to two blood disorders in their early years. Approximately 10% of infants with DS develop transient abnormal myelopoiesis (TAM), a myeloproliferative disorder with an increase in leukocytes and blasts in peripheral blood [1, 2]. While most patients experience spontaneous remission within 6 months, about 10% of patients will have fatal liver dysfunction due to blastic infiltration [1, 3–6] and another 10–20% of patients develop Down syndrome-associated acute megakaryocytic leukemia (DS-AMKL) within 5 years [1, 3, 5, 7–9]. Meta-analyses of clinical reports of TAM and DS-AMKL [7, 10–14] and a case report of monozygotic twins [2, 13, 15, 16] have shown that almost all TAM and DS-AMKL cases have somatic mutations of *GATA-binding protein 1 (GATA1)* gene and that these mutations are essential in the multi-step development process of DS-AMKL.

GATA1 is a representative hematopoietic transcription factor involved in early hematopoiesis and erythro-megakaryocytic cell development [17–27]. Various mutations in exons 2 to 3 of *GATA1* result in the loss of the full-length protein (GATA1fl) and the production of only the short-form protein (GATA1s) translated from the second ATG site, which lacks the amino-terminal activation domain [10, 28]. This means that, regardless of the pattern of the mutation, the resulting protein is always a single alternative form produced even without the mutation, albeit in small amounts. This distinguishes this mutation from other oncogenic mutations.

Despite the obvious necessity for GATA1 mutations in trisomy-21 cells, the quantitative impact of GATA1s protein produced as a result of the mutations has not been fully elucidated. Indeed, although some meta-clinical analyses have shown a significant association between the GATA1s expression levels predicted from the variants and the severity of TAM and the frequency of AMKL [29], the early stage pathogenesis is not fully understood. In particular, it remains unclear whether there is a direct causal relationship beyond correlation between the amount of GATA1s protein, rather than its presence per se, and early hematopoietic cell fate associated with disease-specific blood findings.

An in vitro model using PSCs was reported to be useful for analyzing diseases of early hematopoiesis [30–32]. Of course, it is hard to precisely address if the level of gene expressions in PSC-derived hematopoietic cells be the same in cells of comparable stages in primary disease development during fetal hematopoiesis, but several PSC models of TAM have been already reported to recapitulate a differentiation preference for myelocytes due to *GATA1* mutations and an increase in CD34^+^ immature megakaryoblasts associated with expression level of GATA1s [33–35], which correspond to the features observed in patients. Furthermore, recent study using trisomy-21 PSCs identified an CD34^+^CD43^+^CD11b^-^CD71^+^CD41^+^CD235a^-^ megakaryocytic progenitor population largely responsible for the myeloid proliferation in the absence of GATA1fl [36]. Interestingly, despite being an erythro-megakaryocytic progenitor population, cells in this fraction possessed an expression profile that showed a tendency for myeloid differentiation, which suggested the need for a more detailed analysis of the effect of GATA1s on the nature of progenitors in earlier developmental stages. Current study therefore examined the effects of higher or lower amount of GATA1s protein levels on each lineage cell by additionally induce GATA1s expression in early-stage hematopoietic cells derived from *GATA1* mutant PSCs.

## Materials and methods

### Ethical statement

To establish and use induced pluripotent stem cells (iPSCs), written informed consent was obtained from the guardians of the DS patient (ID: CiRA12345 at Kyoto University and 778 at Hirosaki University) in accordance with the Declaration of Helsinki. The use of human embryonic stem cells (ESCs) in Kyoto University and Tottori University was approved by the Ministry of Education Culture, Sports, Science and Technology of Japan (MEXT). This study was approved by the Ethics Committee and the recombinant DNA Experiments Safely Committee of Kyoto University. All methods were performed in accordance with the relevant guidelines and regulations.

### Cells and cell culture

The cell line Ts21-ES-*GATA1*-WT, in which a human chromosome 21 was transferred into the human ESC line, KhES-1-derived subline, and Ts21-ES-*GATA1s*, in which the GATA1 mutation was introduced into the KhES-1-derived subline and then a human chromosome 21 was transferred into the *GATA1s*-ES, were previously established [33]. TAM-iPS-*GATA1s*, which was generated from the blasts of TAM patients with DS, and TAM-iPS-*GATA1*-WT, in which the *GATA1* mutation of TAM-iPS-*GATA1s* was repaired, were established as described previously [36]. All PSCs were cultured on 0.25 μg/cm^2^ Laminin511-E8 fragment iMatrix-511 silk (Nippi, Tokyo, Japan)-coated culture plates with StemFit AK02 medium (Ajinomoto, Tokyo, Japan). For passage, the cells were dissociated into single cells with 0.5×TrypLE Select (Thermo Fisher Scientific, Waltham, MA, USA) and plated at 265 cells/cm^2^. 10 μM Rock inhibitor Y-27632 (Nacalai Tesque, Kyoto, Japan) was used at the time of the plating, and the medium was exchanged with fresh AK02 medium without Y-27632 the next day.

### Generation of stable Dox-inducible GATA1fl-HA and GATA1s-HA cell lines

The *adeno-associated virus integration site 1* (*AAVS1*) targeting pAAVS1-Tet-on-hGATA1Δex2-HA vector was generated by replacing the CRISPRi cassette of pAAVS1-NDi-CRISPRi (Gen2) purchased from Addgene (plasmid #73498; http://n2t.net/addgene:73498; RRID:Addgene_73498) [37] with C-terminal HA-tagged GATA1Δex2 amplified from the cDNA of the cell line K562 using an In-Fusion HD Cloning Kit (Clontech, Mountain View, CA, USA). The neomycin resistant gene expression cassette was replaced with the hygromycin resistant gene generated by DNA synthesis. The resulting pAAVS1-Tet-on-hGATA1Δex2-HA vector and Cas9/gRNA expressing vector AAVS1 T2 CRISPR in pX330 purchased from Addgene (plasmid #72833; http://n2t.net/addgene:72833; RRID:Addgene_72833) [38] were electroporated into Ts21-ES clones using a NEPA21 electroporator (NEPAGENE, Chiba, Japan). Transfected cells were selected with 50 μg/mL hygromycin (InvivoGen, San Diego, CA, USA). Hygromycin-resistant clones were picked, and successful targeting was confirmed by Sanger sequencing. To generate PB-Tet-on-hGATA1fl-HA vector, the second ATG of the C-terminal HA-tagged GATA1fl fragment amplified from the cDNA of K562 cells was replaced with CTC and cloned into an all-in-one PiggyBac-based Tet-inducible expression cassette vector synthesized in our laboratory. PB-Tet-on-hGATA1-HA vector and PiggyBac transposase vector were electroporated into Ts21-ES-*GATA1s* using the NEPA21 electroporator. Transfected cells were selected with 0.5–1 μg/mL puromycin (InvivoGen).

### Hematopoietic differentiation

**Fig 1:**
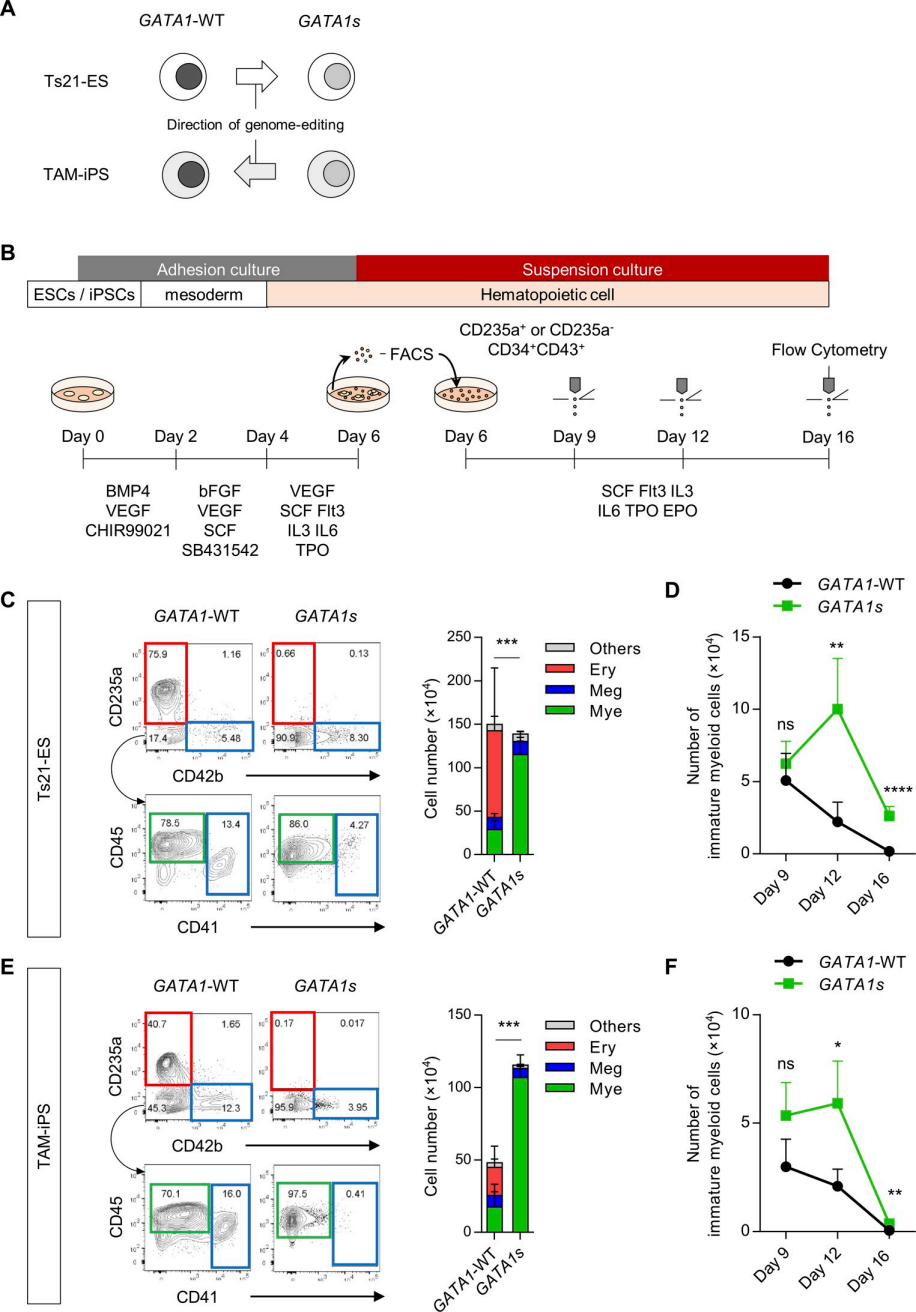
CD235a^-^CD34^+^CD43^+^ early-phase multipotent progenitors recapitulate the hematopoietic features of TAM. (A) Scheme of the *GATA1*-WT and *GATA1s* Ts21-PSC isogenic pairs used in this study. (B) Schematic method for hematopoietic differentiation. CD235a^+^CD34^+^CD43^+^ cells or CD235a^-^CD34^+^CD43^+^ cells (HPCs) were sorted on day 6 and transferred to suspension culture. HPCs were continuously cultured, and cell count and flow cytometry were performed on day 9, day 12 and day 16. (C, E) Representative flow cytometry results and counts of each lineage on day 16 differentiated from the CD235a^-^CD34^+^CD43^+^ population of day 6 (C) Ts21-ES clones and (E) TAM-iPS clones. (D, F) Changes in the number of immature myeloid cells differentiated from the CD235a^-^CD34^+^CD43^+^ population of day 6 (D) Ts21-ES clones and (F) TAM-iPS clones (n = 5 biologically independent experiments for Ts21-ES, n = 4 for TAM-iPS-*GATA1*-WT, n = 3 for TAM-iPS-*GATA1s*). Data are presented as the mean ± SD. *p < 0.05, **p < 0.01, ***p < 0.001, ****p < 0.0001 by two-tailed unpaired Student’s *t*-test for myeloid lineages. Ery, erythrocytic cells; Meg, megakaryocytic cells; Mye, myeloid cells.

The hematopoietic differentiation was performed as previously described (**Fig 1B**) [39, 40]. In brief, undifferentiated PSC colonies were prepared on Laminin511-E8 fragment-coated culture plates with StemFit AK02 medium by seeding single cells or spheroids. When individual colonies reached 750 to 1000 μm in diameter, the culture medium was replaced with Essential 8 medium (Thermo Fisher Scientific) containing 80 ng/mL BMP4 (R&D Systems, Minneapolis, MN, USA), 80 ng/mL VEGF (R&D Systems) and 2 μM GSK-3 inhibitor CHIR99021 (Merck Millipore, Burlington, MA, USA). The cells were cultured at 37°C, 5% CO2 and 5% O2 during differentiation. On day 2, the medium was replaced with Essential 6 medium (Thermo Fisher Scientific) containing 25 ng/mL bFGF (Wako, Osaka, Japan), 80 ng/mL VEGF, 50 ng/mL SCF (R&D Systems) and 2 μM SB431542 (Sigma-Aldrich, St. Louis, MO, USA). On day 4, the medium was replaced with Stemline^®^ Ⅱ medium (Sigma-Aldrich) containing 80 ng/mL VEGF, 50 ng/mL SCF, 50 ng/mL Flt-3 Ligand (R&D Systems), 50 ng/mL IL-3 (R&D Systems), 50 ng/mL IL-6 (R&D Systems) and 5 ng/mL thrombopoietin (TPO, R&D Systems). On day 6, the cultured cells were gently dissociated with 0.5×TrypLE Select and filtered through a 40 μm cell strainer. Hematopoietic progenitor cells (HPCs) sorted by FACS Aria Ⅱ (BD Biosciences, San Jose, CA, USA) were cultured at a density of 1×10^4^ cells per well in 24-well plate with Stemline^®^ Ⅱ medium containing 50 ng/mL SCF, 50 ng/mL Flt-3 Ligand, 50 ng/mL IL-3, 50 ng/mL IL-6, 5 ng/mL TPO and 2 U/mL erythropoietin (EPO, Merck Millipore). The same amount of medium was added every 2 days, and the cells were re-seeded at a density of 2×10^4^ cells per well in a 24-well plate on day 9 and day 12.

### Cell sorting and flow cytometric analyses

The isolation of HPCs on day 6 and subsequent flow cytometric analysis were performed by using a FACS Aria Ⅱ (BD Biosciences). The antibodies used are described in **Table 1**. Collected cells were counted using C-chip (NanoEnTek, Seoul, Korea) or Countess^®^ Ⅱ FL automated cell counter (Thermo Fisher Scientific) and stained in PBS containing 2% FBS for 20 minutes on ice. Samples were analyzed using FlowJo software (FlowJo LLC, Ashland, OR, USA).

**Table 1:** Antibodies used for flow cytometric analysis.

Antigen	Fluorochrome	Clone	Clonality	Source	Catalog #
CD309 (KDR)	Alexa Fluor^®^ 647	7D4-6	monoclonal	Biolegend	359910
CD235a	BV421	GA-R2 (HIR2)	monoclonal	BD Biosciences	562938
CD34	Brilliant Violet 605	581	monoclonal	Biolegend	343529
CD43	PE/Cy7	CD43-10G7	monoclonal	Biolegend	343208
CD45	FITC	2D1	monoclonal	Biolegend	368508
CD42b	PE	HIP1	monoclonal	Biolegend	303906
CD71	APC	CY1G4	monoclonal	Biolegend	334108
CD33	PE/Cy7	WM53	monoclonal	Biolegend	303434
CD41	APC/Cy7	HIP8	monoclonal	Biolegend	303716
CD11b	PerCP/Cy5.5	ICRF44	monoclonal	Biolegend	301328

(Biolegend, San Diego, CA, USA)

List of antibodies used for flow cytometric analysis.

### Immunoblotting

To confirm the expression of Dox-inducible GATA1 protein, protein was extracted from human PSCs treated with or without 1 μg/mL Dox for 24 hours with RIPA buffer (Wako) supplemented with 2% protease inhibitor cocktail (Nacalai, Kyoto, Japan). Each sample was separated by 10% sodium dodecyl sulfate polyacrylamide gel electrophoresis and transferred to PVDF membranes (Merck Millipore). The membrane was blocked with 5% dry milk and incubated with an anti-GATA1 primary antibody (CST #4589, 1/1,000, Danvers, MA, USA) overnight at 4°C. The membrane was then incubated with anti-rabbit IgG, HRP-linked secondary antibody (CST #7074, 1/5,000) for 1 hour at room temperature. To confirm the amount of loaded protein, the membrane was stripped with WB stripping solution strong (Nacalai) and probed with ꞵ-actin (13E5) rabbit mAb (CST #4970, 1/2,000). Signals were detected with Chemi-Lumi One Super (Nacalai) and scanned with ImageQuant LAS 4000 (GE Healthcare, Chicago, IL, USA).

### Statistical analyses

Statistical analyses were performed with GraphPad Prism 6 (GraphPad Soft, La Jolla, CA, USA). Results are shown as the mean ± SD and compared with the unpaired Student’s *t*-test.

## Results

### CD235a^-^CD34^+^CD43^+^ early-phase multipotent progenitors recapitulate the hematopoietic features of TAM

In order to precisely analyze the effect of *GATA1* genotype on the hematopoietic differentiation process, we prepared two sets of isogenic PSC pairs with trisomy of chromosome 21. One pair was human ESCs transferred chromosome 21 (Ts21-ES-*GATA1*-WT) and the same line with *GATA1* mutation introduced (Ts21-ES-*GATA1s*) [33]. The other pair was iPSCs (TAM-iPS-*GATA1s*) established from the blasts of a TAM patient with DS and with the *GATA1* mutation that repaired (TAM-iPS-*GATA1*-WT) [36] (**Fig 1A**). To compare these isogenic pairs, we conducted hematopoietic differentiation (**Fig 1B**).

In our hematopoietic differentiation system, KDR^-^CD34^+^CD43^+^ early-phase HPCs arose from both *GATA1*-WT and *GATA1s* strains on day 6 of the initial differentiation and were divided into two fractions: CD235a positive and negative, respectively (**S1A Fig**). From the early period of the secondary culture after sorting, CD235a^+^ HPCs in the *GATA1-*WT strains already showed commitment to erythroid (CD235a^+^CD42b^-^) cells on day 9 (**S1B, S1C, S1E and S1F Fig**) and almost no production of immature myeloid cells (CD34^+^CD235a^-^CD41^-^CD42b^-^CD45^+^) (**S1D and S1G Fig**). In contrast, CD235a^-^ HPCs produced immature myeloid cells (**S1D and S1G Fig**) and finally differentiated into all erythroid, megakaryocytic (CD235a^-^CD41^+^) and myeloid (CD235a^-^CD41^-^CD42b^-^CD45^+^) lineage cells on day 16 (**Fig 1C and 1E**), which suggested the multipotency of the later subpopulation in our hematopoietic system. To dissect the spatiotemporal impact of *GATA1* mutation on each lineage cell fate, we applied the KDR^-^CD235a^-^CD34^+^CD43^+^ fraction to subsequent cultures as early-phase multipotent HPCs (hereafter called “early HPCs”).

Compared to the *GATA1*-WT strains, early HPCs in *GATA1s* strains produced few erythroid lineage cells and much more myeloid lineage cells (**Fig 1C and 1E**). Of note, while immature myeloid cells derived from the *GATA1*-WT strains continued to decrease with time, those from the *GATA1s* strains increased until day12 of the culture and were maintained significantly longer than in the *GATA1*-WT strains thereafter (**Fig 1D and 1F**). Both strains gave rise to megakaryocytic lineage cells (**Fig 1C and 1E**), which is consistent with previous studies that showed GATA1fl is not essential for specification into megakaryocytes, unlike erythrocytes [22, 33–35, 41, 42]. Taken together, these data indicated that early HPCs can recapitulate the hematopoietic features of TAM [1].

### Establishment of Doxycycline-inducible GATA1s- or GATA1fl-expressing clones

**Fig 2:**
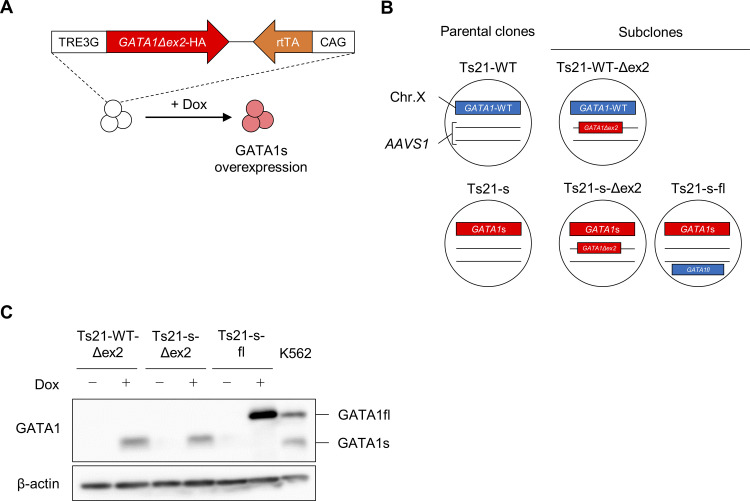
Establishment of Dox-inducible GATA1s or GATA1fl Ts21-ES cells. (A) Scheme of the Dox-inducible GATA1s. (B) Parental clones and generated GATA1s or GATA1fl Dox-inducible subclones. The Dox-inducible GATA1s construct was knocked into *AAVS1* locus with the CRISPR-Cas9 system, and the Dox-inducible GATA1fl construct was transduced by the PiggyBac system. (C) Western blot analysis of GATA1s and GATA1fl expression in untreated ESCs and ESCs treated with 1 μg/ml Dox for 24 h. K562 was used as the positive control.

Previous studies have reported that GATA1s is not just the cause of increased myelocytes in TAM, but also that higher expression levels correlate with severe disease groups [29, 41]. On the other hand, the incidence of DS-AMKL, which is an oncogenic blast proliferation derived from megakaryocytic progenitors, correlates with a lower expression of GATA1s, suggesting that GATA1s has different effects on the myeloid and megakaryocytic lineages in the absence of GATA1fl [7, 29]. To clarify this spatiotemporal quantitative effect of GATA1s protein on the nature of multipotent progenitors and each lineage cell type, we next analyzed the differentiation properties of *GATA1s* strains introduced with Dox-inducible GATA1s expression cassettes (**Fig 2A and S2A and S2B Fig**). Additionally, we generated *GATA1*-WT strains with Dox-inducible GATA1s expression cassettes and *GATA1s* strains in which we added the Dox-inducible GATA1fl expression cassettes to evaluate the emergence and rescue of disease phenotypes, respectively (**S2C Fig and Fig 2B**). The insertion of the GATA1s expression cassette was confirmed by genomic PCR (**S2B Fig**), and protein expressions induced by Dox treatment were confirmed by western blotting analyses (**Fig 2C**). Karyotypes of each clones was confirmed by Q-banding analysis (**S3A–S3E Fig**). To confirm whether there is reproducibility beyond the clones, we also generated corresponding subclones in TAM-iPS clones (**S4A Fig**), and confirmed karyotypes and Dox-inducible expression of GATA1 protein (**S4B–S4G Fig**).

### GATA1s protein acts to quantitatively sustain immature myeloid cells in competition with GATA1fl

**Fig 3:**
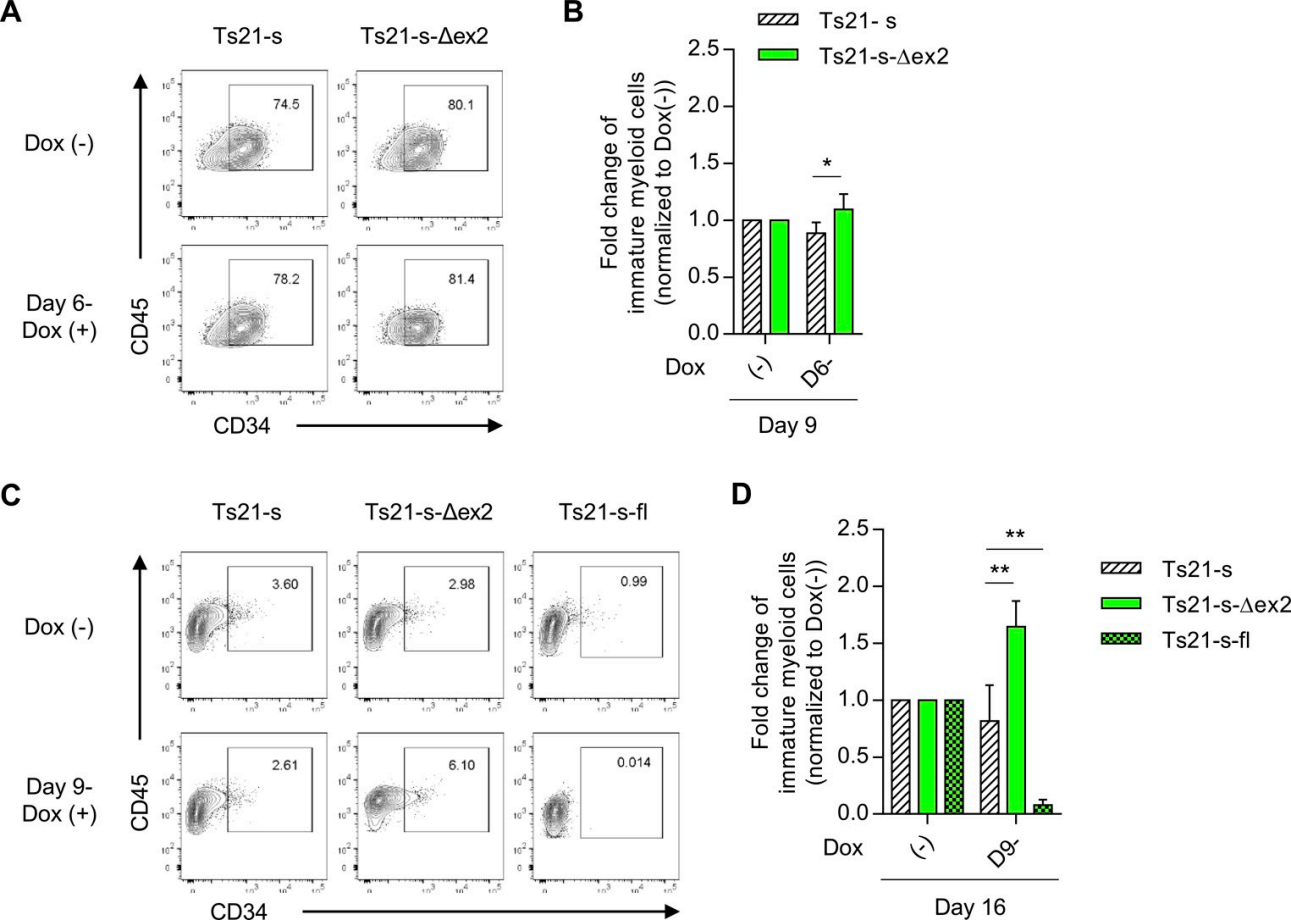
Quantitative increase of GATA1s in early-phase increases myeloid commitment and enhances the maintenance of immature myeloid cells. (A) Representative flow cytometry of staining for CD34 and CD45 among myeloid cells on day 9. Upper panels indicate the Dox-untreated sample and lower panels indicate the Dox-treated sample from day 6 for each clone. (B) Fold changes of immature myeloid cells over each untreated sample on day 9. (C) Representative flow cytometry of staining for CD34 and CD45 among myeloid cells on day 16 with or without Dox treatment from day 9. (D) Fold changes of immature myeloid cells over each untreated sample on day 16 (n = 5 biologically independent experiments for Ts21-s and Ts21-s-Δex2, n = 3 for Ts21-s-fl). Data are presented as the mean ± SD. *p < 0.05, **p < 0.01 vs. Ts21-s under the same treatment by two-tailed unpaired Student’s *t*-test.

Using the series of modified cells, we examined the quantitative effects of GATA1s by lineage. GATA1s overexpression in early HPCs on day 6 significantly increased commitment into myeloid lineage (**Fig 3A and 3B**). Moreover, overexpression from day 9 of the differentiation, when immature myeloid progenitors had already appeared in culture (**Fig 1D**), also significantly increased the number of immature myeloid progenitors (**Fig 3C and 3D**). Considering that GATA1fl deficiency itself led to an increase in myeloid cells even without exogenous *GATA1s* expression (**Fig 1D**), these results suggested that GATA1s leads to a further proliferation of the myeloid lineage brought about by the loss of GATA1fl by sustaining committed progenitors. Consistent with this result, we observed that overexpression of GATA1s tended to increase the number of colonies containing non-megakaryocytic (non-Mk) cells in colony-forming unit assay of megakaryocytic progenitors (CFU-Mk) (**S5A, S5B and S5D Fig**) and larger non-Mk colonies was seen in GATA1s overexpressed samples (**S5E Fig**) as previously reported [30]. In TAM-iPS-*GATA1s* derived clones, due to differences in the differentiation properties, it was not possible to detect increase myeloid commitment by quantitative increase of GATA1s (**S6A and S6B Fig**), but there was tendency toward enhanced maintenance of immature myeloid cells (**S6C and S6D Fig**). These results are consistent with the exacerbation of myeloproliferation in patients with a higher expression of GATA1s. Similar results were obtained in *GATA1*-WT strains introduced with *GATA1s* (**S7A and S7B Fig**) and similar result was obtained for TAM-iPS-*GATA1*-WT derived clone (**S8A and S8B Fig**). Whereas, the opposite was observed in *GATA1s* strains that overexpressed GATA1fl (**Fig 3C and 3D**), demonstrating that GATA1s and GATA1fl competitively increase and decrease myeloid lineages.

### GATA1s protein has conflicting effects on megakaryocyte commitment and persistence in the absence of GATA1fl

**Fig 4:**
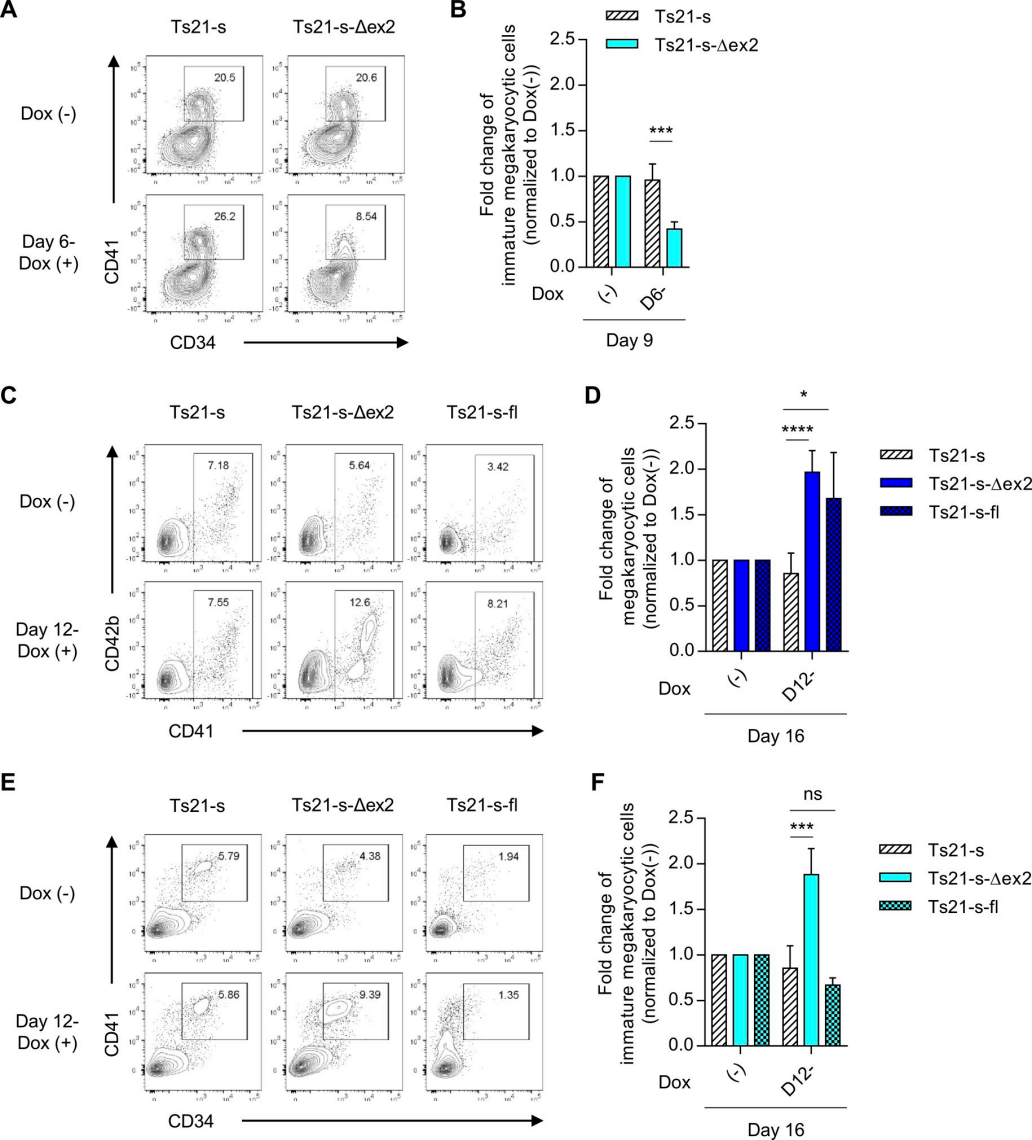
Quantitative increase of GATA1s in early-phase suppresses megakaryocytic differentiation and in later-phase increases the persistence of immature megakaryocytic cells. (A) Representative flow cytometry of staining for CD34 and CD41 on day 9. Upper panels indicate the Dox-untreated sample and lower panels indicate the Dox-treated sample from day 6 for each clone. (B) Fold changes of immature megakaryocytic cells over each untreated sample on day 9. (C) Representative flow cytometry of staining for CD41 and CD42b on day 16 with or without Dox treatment from day 12. (D) Fold changes of megakaryocytic cells over each untreated sample on day 16. (E) Representative flow cytometry of staining for CD34 and CD41 on day 16 with or without Dox treatment from day 12. (F) Fold changes of immature megakaryocytic cells over each untreated sample on day 16 (n = 5 biologically independent experiments for Ts21-s and Ts21-s-Δex2, n = 3 for Ts21-s-fl). Data are presented as the mean ± SD. *p < 0.05, ***p < 0.001, ****p < 0.0001 vs. Ts21-s under the same treatment by two-tailed unpaired Student’s *t*-test.

Contrary to the correlation with myeloproliferation seen in TAM, meta-clinical analyses on the impact of *GATA1* mutation in DS-AMKL are somewhat paradoxical. Although almost all DS-AMKL patients have a *GATA1* mutation, some studies have shown that an increased expression of GATA1s is inversely associated with the risk of DS-AMKL [29]. We therefore evaluated the spatiotemporal effects of GATA1s on megakaryocytic lineage, a potential origin of DS-AMKL, following differentiation. GATA1s overexpression in early HPCs significantly reduced megakaryocytic commitment in *GATA1s* strains (**Fig 4A and 4B**). Similar results was obtained with TAM-iPS-*GATA1s* derived clone (**S9A and S9B Fig**). Consistent with this result, we observed that the overexpression of GATA1s significantly reduced the total number of CFU-Mk (**S5A–S5C Fig**). Furthermore, an effect of GATA1s overexpression was observed in *GATA1s* strains but not in *GATA1*-WT strains (**S7C and S7D Fig**) and in TAM-iPS-*GATA1*-WT derived clone (**S8C and S8D Fig**), suggesting that the effects on megakaryocytic lineage are counteracted by endogenous GATA1fl, even at high concentrations of GATA1s. On the other hand, unexpectedly, GATA1fl overexpression did not restore the megakaryocytic differentiation of *GATA1s* strains, but rather reduced it as in the case of GATA1s overexpression (**S10A, S10B, S11 and S11B Figs**). Because the predominant restoration of erythroid differentiation was observed at this time (**S10C–S10E and S11C–S11E Figs**), these results indicated that GATA1fl at the endogenous expression level is important for the commitment to both erythroid and megakaryocytic lineages, but a higher expression at this stage leads to a significant bias towards erythroid commitment due to its essential role in erythropoiesis, which consequently suppresses megakaryocyte commitment.

The inhibitory effects of GATA1s on megakaryocytic commitment could explain the lower risk of DS-AMKL progression in cases of high GATA1s expression among TAM patients [29]. Nevertheless, it is still clinically evident that *GATA1* mutations are by far the most important risk factor for developing DS-AMKL, even in patients with a high expression of GATA1s [1, 2]. These facts led us to examine if there is another cause of the accumulation of immature megakaryocytes that could be responsible for DS-AMKL even in GATA1s high-expressing cells with suppressed commitment. Indeed, we found the overexpression of either GATA1s and GATA1fl significantly increased the percentage of total megakaryocytes in *GATA1s* strains after day 12 of the differentiation (**Fig 4C and 4D**). However, when focusing on immature megakaryocytic progenitor cells, GATA1s overexpression had a significantly increased CD34^+^CD41^+^ subpopulation, but GATA1fl overexpression did not. (**Fig 4E and 4F**). In TAM-iPS-*GATA1s* derived clone, although there was no significant difference in total megakaryocytes, there was a trend toward an increase (**S9C and S9D Fig**). Whereas, when we focused on immature megakaryocytic cells, we found that the overexpression of GATA1s in megakaryocytic progenitors on later stage significantly increased the persistence of immature megakaryocytic cells, but GATA1fl overexpression did not (**S9E and S9F Fig**). These results indicated that GATA1s works to maintain immature cells in megakaryocytic lineage as well as myeloid lineage, but unlike the myeloid lineage, the overexpression of GATA1s in the *GATA1*-WT strain did not have any effect on immature megakaryocytic cells (**S7E, S7F, S8E and S8F Figs**). Therefore, the effects of higher GATA1s expression on the maintenance of mutant strain-derived megakaryocytic progenitors are dependent on differences in the responsiveness of the target cells to GATA1s protein, which are conferred by the mutation itself.

## Discussion

The exclusive expression of GATA1s protein as a result of *GATA1* mutations is an essential process for the onset of both TAM and DS-AMKL. Even though blasts in patients in most cases have been found to be a heterogeneous population with a variety of *GATA1* mutations at different expression levels, no study has experimentally examined how the intensity of the gene expression contributes to the pathologies of both diseases. Focusing on this point, we clarified how the spatiotemporal shift of GATA1s protein expression affects the progenitor cells from which both diseases originate by using a PSC model and stepwise hematopoietic differentiation. We successfully observed the quantitative impact of the GATA1s expression level on each stage of each lineage by utilizing a Dox-inducible expression system.

PSC-based studies can reveal new effects of mutant genes that cannot be elucidated by studies using patient primary cells after the disease onset or cell lines that are already addicted to the mutations themselves. Moreover, with respect to DS, there is no suitable mouse model that replicates the phenotypes of human trisomy-21. While previous studies including the over-expression of GATA1s in fetal liver progenitor cells of *Gata1*^*ΔN*^ mice and cord blood CD34^+^ hematopoietic progenitor cells have reported the GATA1s-dependent expansion of *GATA1* mutant cells in myeloid and megakaryocytic lineages [41, 43], our study distinguished the effects of GATA1s on the commitment and proliferation of the myeloid and megakaryocytic lineages in the absence of GATA1fl by focusing on the progenitor cells which correspond to common myeloid progenitors, originally defined as an origin of both granulocyte/macrophage progenitors and megakaryocyte/erythrocyte progenitors. Specifically, we found that commitment to megakaryocytes at the early HPC stage were significantly reduced by elevated GATA1s expression, and only in the absence of GATA1fl were the megakaryocyte progenitors maintained in response to GATA1s expression levels. These mutation- and differentiation stage-specific reversal effects contrasted the results regarding myeloid lineage, where GATA1s simply sustained and increased progenitor cells in competition with GATA1fl.

Two hypotheses may explain why once committed megakaryocytic progenitors acquire the ability to proliferate in response to GATA1s like myeloid progenitors only under conditions without GATA1fl. First, some additional genetic or epigenetic modifications that occur during tumorigenesis might confer GATA1s-responsive growth characteristics. Alternatively, GATA1fl deficiency itself might provide intracellular signaling for the perturbation. Indeed, a previous study using trisomy-21 PSCs revealed that the expression profile of a GATA1fl-deficient megakaryocytic progenitor subpopulation responsible for myeloproliferation was biased toward the myeloid lineage [36]. Therefore, GATA1s could hijack the myeloid mechanism to promote the proliferation of megakaryocytic progenitors. Further study of this hypothesis using methods that directly examine access of the GATA1 protein to genomic DNA, such as electrophoretic mobility shift assays and chromatin immunoprecipitation, are needed. Such studies could also reveal new molecular mechanisms, by which the higher expression of GATA1s suppresses megakaryocytic commitment in early HPCs.

**Fig 5:**
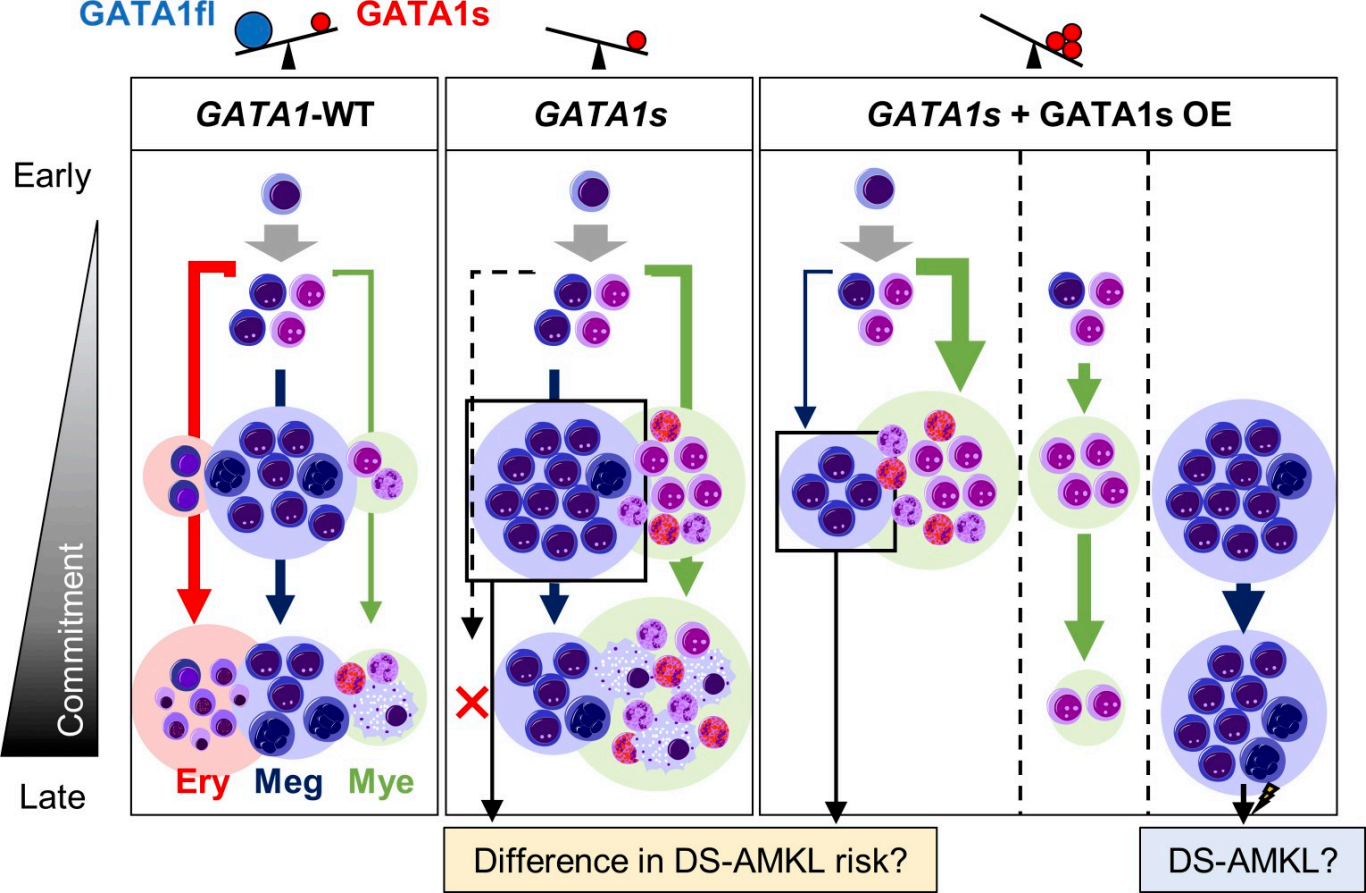
Graphical abstract of *GATA1*-WT, *GATA1s* and the effects of GATA1s overexpression on *GATA1s* strain. With wild-type *GATA1* (*GATA1*-WT), which expresses both the full length (GATA1fl) and short form (GATA1s) of GATA1 protein, all erythrocytic (Ery), megakaryocytic (Meg) and myeloid (Mye) lineages are produced. In the case of *GATA1s* mutation, erythroid differentiation is markedly impaired and myeloid cells are increased. With the additional overexpression of GATA1s, *GATA1s* mutation suppresses megakaryocytic differentiation and increases myeloid commitment. In addition, the persistence of immature megakaryocytic cells is enhanced in the later phase.

Collectively, our results suggested that although *GATA1* mutant cells cause the increase in myeloid and megakaryocytic progenitors regardless of the intensity of GATA1s expression, the pathways vary with their expression levels **(Fig 5)**. This model provides an explanation for the paradoxical clinical features in which higher and lower GATA1s expressions are inversely correlated with the severity of TAM and development of DS-AMKL among patients with TAM even though *GATA1* mutations are the definitive etiology of both diseases. Future in vitro and in vivo studies are expected to provide more definitive evidence for this model.

## Supporting information

S1 Fig Characterization of CD235a^+^CD34^+^CD43^+^ cells compared with CD235a^-^CD34^+^CD43^+^ cells. (A) Gating strategy used to sort CD235a^+^CD34^+^CD43^+^ and CD235a^-^CD34^+^CD43^+^ HPCs on day 6. (B-C, E-F) Representative flow cytometric analysis and cell number of each population on day 9 compared with the CD235a^+^CD34^+^CD43^+^ (235a^+^) and CD235a^-^CD34^+^CD43^+^ (235a^-^) populations of (B, C) Ts21-ES-*GATA1*-WT and (E, F) TAM-iPS-*GATA1*-WT. (D, G) Changes in the number of immature myeloid cells compared with the CD235a^+^CD34^+^CD43^+^ and CD235a^-^CD34^+^CD43^+^ populations differentiated on day 6 of (D) Ts21-ES-*GATA1*-WT and (G) TAM-iPS-*GATA1*-WT (n = 3 biologically independent experiments for CD235a^+^CD34^+^CD43^+^ of Ts21-ES-*GATA1*-WT and TAM-iPS-*GATA1*-WT, n = 5 for CD235a^-^CD34^+^CD43^+^ of Ts21-ES-*GATA1*-WT and n = 4 for CD235a^-^CD34^+^CD43^+^ of TAM-iPS-*GATA1*-WT). Data are presented as the mean ± SD. *p < 0.05, **p < 0.01, ***p < 0.001 by two-tailed unpaired Student’s *t*-test. Ery, erythrocytic cells; Meg, megakaryocytic cells; Mye, myeloid cells.
(TIFF)

S2 Fig Establishment of GATA1 isoform Dox-inducible clones. (A) Schematic overview of the *AAVS1* targeting strategy by CRISPR-Cas9 to generate Dox-inducible GATA1s for Ts21-ES lines. (B) Genomic PCR to confirm the integration of the Dox-inducible GATA1s cassette. Expected fragment size: integration of Dox-inducible GATA1Δex2-HA, 8510 bp; no integration, 1956 bp. (C) Scheme of Dox-inducible GATA1fl and PiggyBac vector for Dox-inducible GATA1fl. The second ATG was replaced with CTC to express only GATA1fl.
(TIFF)

S3 Fig Karyotyping of parental Ts21-ES clones and Dox-inducible GATA1s or GATA1fl knock-in subclones. (A-E) Representative Q-banding karyotypes of (A) Ts21-ES-*GATA1*-WT (Ts21-WT), (B) Ts21-ES-*GATA1s* (Ts21-s), (C) Ts21-WT-Δex2, (D) Ts21-s-Δex2 and (E) Ts21-s-fl.
(TIFF)

S4 Fig Establishment of Dox-inducible GATA1s or GATA1fl TAM-iPS cells. (A) Parental clones and generated GATA1s or GATA1fl Dox-inducible subclones. The Dox-inducible GATAs construct was knocked into *AAVS1* locus with CRISPR-Cas9 system, and the Dox-inducible GATA1fl construct was transduced by the PiggyBac system. (B-F) Representative Q-banding karyotypes of (B) TAM-iPS-*GATA1*-WT (TAM-WT), (C) TAM-iPS-*GATA1s* (TAM-s), (D) TAM-WT-Δex2, (E) TAM-s-Δex2 and (F) TAM-s-fl. (G) Western blot analysis of GATA1s and GATA1fl expression in untreated iPSCs and iPSCs treated with 1 μg/mL Dox for 24 h. K562 was used as the positive control.
(TIFF)

S5 Fig CFU-Mk is significantly decreased by GATA1s overexpression in *GATA1s* strains. (A) Representative images of each types of colonies in colony-forming unit assay of megakaryocytic progenitors. (B-D) Numbers of CFUs resulting from 2,500 CD235a^-^CD34^+^CD43^+^ cells on day 6 with or without Dox treatment, (B) total, (C) total of CFU-Mk and (D) total of mixed CFU-Mk/ non-Mk and non-Mk (n = 3 biologically independent experiments for Ts21-WT and Ts21-s-Δex2 and n = 4 for Ts21-s). (E) Representative images of non-Mk colonies observed in Dox-untreated and Dox-treated Ts21-s-Δex2. Scale bars: 100 μm. Data are presented as the mean ± SD. **p < 0.01 vs. untreated sample of each clones by two-tailed unpaired Student’s *t*-test.
(TIFF)

S6 Fig Quantitative increase of GATA1s in TAM-iPS-*GATA1s* derived cells shows tendency to enhance the sustain of immature myeloid cells. (A) Representative flow cytometry of staining for CD34 and CD45 among myeloid cells on day 9. Upper panels indicate the Dox-untreated sample and lower panels indicate the Dox-treated sample from day 6 for each clone. (B) Fold changes of immature myeloid cells over each untreated sample on day 9. (C) Representative flow cytometry of staining for CD34 and CD45 among myeloid cells on day 12 with or without Dox treatment from day 9. (D) Fold changes of immature myeloid cells over each untreated sample on day 16 (n = 3 biologically independent experiments). Data are presented as the mean ± SD. ns vs. TAM-s under the same treatment by two-tailed unpaired Student’s *t*-test.
(TIFF)

S7 Fig Overexpression of GATA1s has little effect on immature megakaryocytic cells in the presence of GATA1fl. (A) Representative flow cytometry of staining for CD34 and CD45 among myeloid cells on day 12. Upper panels indicate the Dox-untreated sample and lower panels indicate the Dox-treated sample from day 9. (B) The fold changes of immature myeloid cells over each untreated sample on day 12 and day 16. (C, E) Representative flow cytometry of staining for CD34 and CD41 (C) on day 9 with or without Dox treatment from day 6 and (E) on day 16 with or without Dox treatment from day 12. (D, F) The fold changes of immature megakaryocytic cells over each untreated sample (D) on day 9 and (F) on day 16 (n = 4 biologically independent experiments for Ts21-WT and n = 3 for Ts21-WT-Δex2). Data are presented as the mean ± SD. *p < 0.05 vs. Ts21-WT under same treatment by two-tailed unpaired Student’s *t*-test.
(TIFF)

S8 Fig Over expression of GATA1s also has little effect on immature megakaryocytic cells of TAM-iPS-*GATA1*-WT derived cells. (A) Representative flow cytometry of staining for CD34 and CD45 among myeloid cells on day 12. Upper panels indicate the Dox-untreated sample and lower panels indicate the Dox-treated sample from day 9. (B) The fold change of immature myeloid cells over untreated sample on day 12 and day 16. (C, E) Representative flow cytometry of staining for CD34 and CD41 (C) on day 9 with or without Dox treatment from day 6 and € on day 16 with or without Dox treatment from day 9. (D, F) The fold changes of immature megakaryocytic cells over each untreated sample (D) on day 9 and (F) on day 16 (n = 3 biologically independent experiments). Data are presented as the mean ± SD. ***p < 0.001 vs. Ts21-WT under same treatment by two-tailed unpaired Student’s *t*-test.
(TIFF)

S9 Fig Conflicting effects of quantitative increase of GATA1s on commitment and persistence is also observed in TAM-iPS-GATA1s derived cells. (A) Representative flow cytometry of staining for CD34 and CD41 on day 9. Upper panels indicate the Dox-untreated sample and lower panels indicate the Dox-treated sample from day 6 for each clone. (B) Fold changes of immature megakaryocytic cells over each untreated sample on day 9. (C) Representative flow cytometry of staining for CD41 and CD42b on day 16 with or without Dox treatment from day 9. (D) Fold changes of megakaryocytic cells over each untreated sample on day 16. (E) Representative flow cytometry of staining for CD34 and CD41 on day 16 with or without Dox treatment from day 9. (F) Fold changes of immature megakaryocytic cells over each untreated sample on day 16 (n = 3 biologically independent experiments). Data are presented as the mean ± SD. *p < 0.05, **p < 0.01 by two-tailed unpaired Student’s *t*-test.
(TIFF)

S10 Fig Erythroid differentiation defect of *GATA1s* is remarkably recovered by GATA1fl overexpression in the early stage. (A) Representative flow cytometry of staining for CD34 and CD41 on day 9. Upper panels indicate the Dox-untreated sample and lower panels indicate the Dox-treated sample from day 6. (B) The fold changes of immature megakaryocytic cells over each untreated sample on day 9. (C) Representative flow cytometry of staining for CD71 and CD235a on day 16 with or without Dox treatment from day 6. (D) Average number of CD235a^+^ erythrocytic cells on day 16 (n = 5 biologically independent experiments for Ts21-s and n = 3 for Ts21-s-fl). (E) May-Giemsa staining of Ts21-s-fl on day 16 with or without Dox treatment from day 6. Scale bars: 50 μm. Data are presented as the mean ± SD. **p < 0.01, ***p < 0.001 vs. Ts21-s under same treatment by two-tailed unpaired Student’s *t*-test.
(TIFF)

S11 Fig Erythroid differentiation defect is also remarkably recovered in TAM-iPS-*GATA1s* derived cells by GATA1fl overexpression. (A) Representative flow cytometry of staining for CD34 and CD41 on day 9. Upper panels indicate the Dox-untreated sample and lower panels indicate the Dox-treated sample from day 6. (B) The fold changes of immature megakaryocytic cells over each untreated sample on day 9. (C) Representative flow cytometry of staining for CD71 and CD235a on day 16 with or without Dox treatment from day 6. (D) Average number of CD235a^+^ erythrocytic cells on day 16 (n = 3 biologically independent experiments). (E) May-Giemsa staining of TAM-s-fl on day 16 with or without Dox treatment from day 6. Scale bars: 50 μm. Data are presented as the mean ± SD. **p < 0.01, ***p < 0.001 vs. TAM-s under same treatment by two-tailed unpaired Student’s *t*-test.
(TIFF)

S12 Fig The original uncropped and unadjusted gel and blot images. (A) The original image of electrophoretic gel of **S2B Fig**. lane 1, Marker; lane 2, water; lane 3, Ts21-WT; lane 4, Ts21-WT-Δex2; lane 5, Ts21-s; lane 6, Ts21-s-Δex2; lane 7–8, not shown. (B, C) Original uncut gel images of western blot analysis. (B) Ts21-ES clones on **Fig 2C**; lane 1–7, not shown; lane 8, Marker; lane 9, Ts21-WT-Δex2 Dox (-); lane 10, Ts21-WT-Δex2 Dox (+); lane 11, Ts21-s-Δex2 Dox (-); lane 12, Ts21-s-Δex2 Dox (+); lane 13, Ts21-s-fl Dox (-); lane 14, Ts21-s-fl Dox (+); lane 15, K562. (B) TAM-iPS clones on **S4G Fig**; lane 1, Marker; lane 2, TAM-WT-Δex2 Dox (-); lane 3, TAM-WT-Δex2 Dox (+); lane 4, TAM-s-Δex2 Dox (-); lane 5, TAM-s-Δex2 Dox (+); lane 6, TAM-s-fl Dox (-); lane 7, TAM-s-fl Dox (+); lane 8, K562.
(TIFF)

S1 File (DOCX)
