# Supplementary figures and images for: Pluripotent stem cell model of early hematopoiesis in Down syndrome reveals quantitative effects of short-form GATA1 protein on lineage specification
Source: PLoS One. 2021 Mar 29;16(3):e0247595. doi: 10.1371/journal.pone.0247595 (PMC8007000; doi:10.1371/journal.pone.0247595)

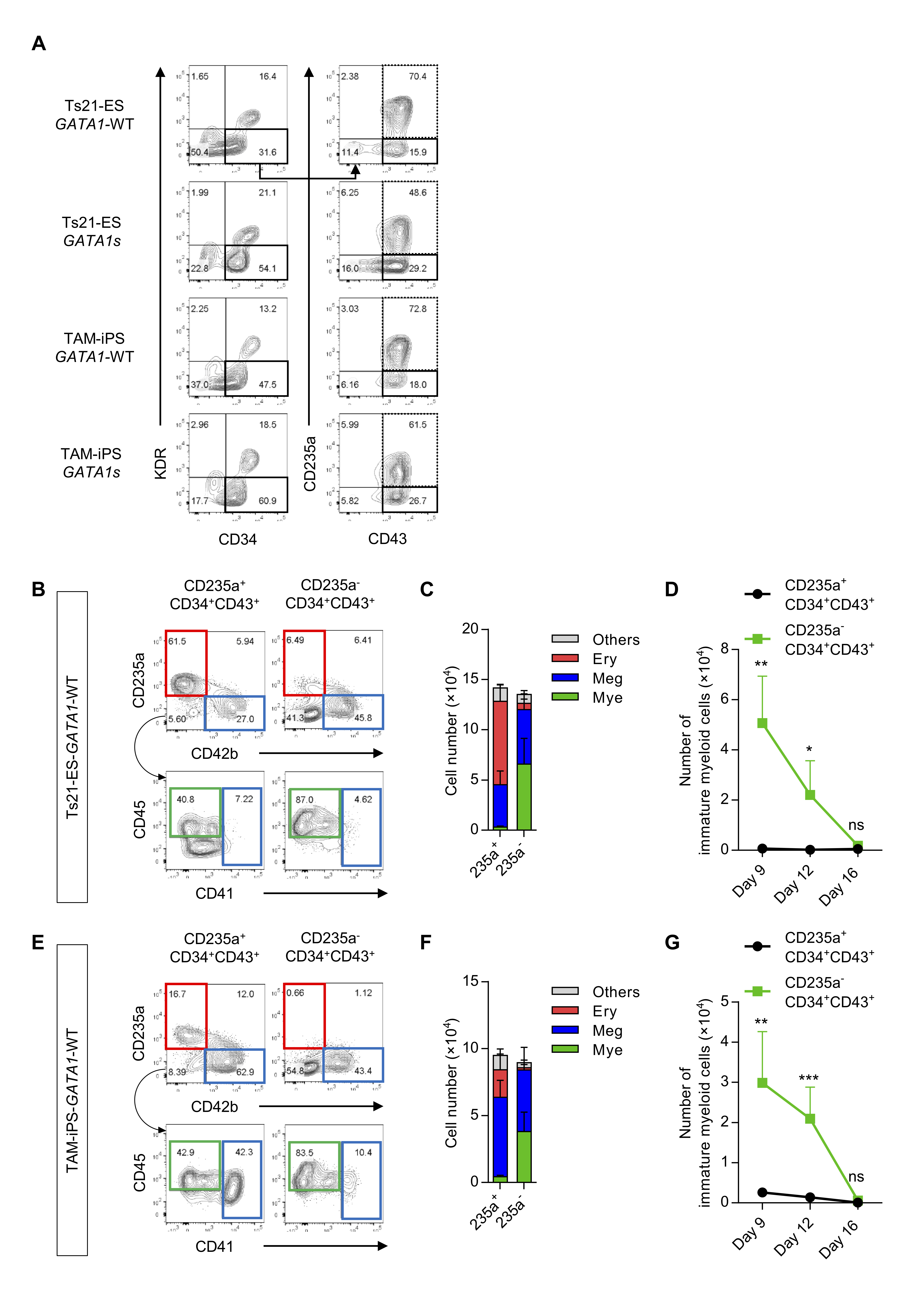

Supplement: S1 Fig — (A) Gating strategy used to sort CD235a+CD34+CD43+ and CD235a-CD34+CD43+ HPCs on day 6. (B-C, E-F) Representative flow cytometric analysis and cell number of each population on day 9 compared with the CD235a+CD34+CD43+ (235a+) and CD235a-CD34+CD43+ (235a-) populations of (B, C) Ts21-ES-GATA1-WT and (E, F) TAM-iPS-GATA1-WT. (D, G) Changes in the number of immature myeloid cells compared with the CD235a+CD34+CD43+ and CD235a-CD34+CD43+ populations differentiated on day 6 of (D) Ts21-ES-GATA1-WT and (G) TAM-iPS-GATA1-WT (n = 3 biologically independent experiments for CD235a+CD34+CD43+ of Ts21-ES-GATA1-WT and TAM-iPS-GATA1-WT, n = 5 for CD235a-CD34+CD43+ of Ts21-ES-GATA1-WT and n = 4 for CD235a-CD34+CD43+ of TAM-iPS-GATA1-WT). Data are presented as the mean ± SD. *p < 0.05, **p < 0.01, ***p < 0.001 by two-tailed unpaired Student’s t-test. Ery, erythrocytic cells; Meg, megakaryocytic cells; Mye, myeloid cells. (TIFF) [file pone.0247595.s001.tiff]

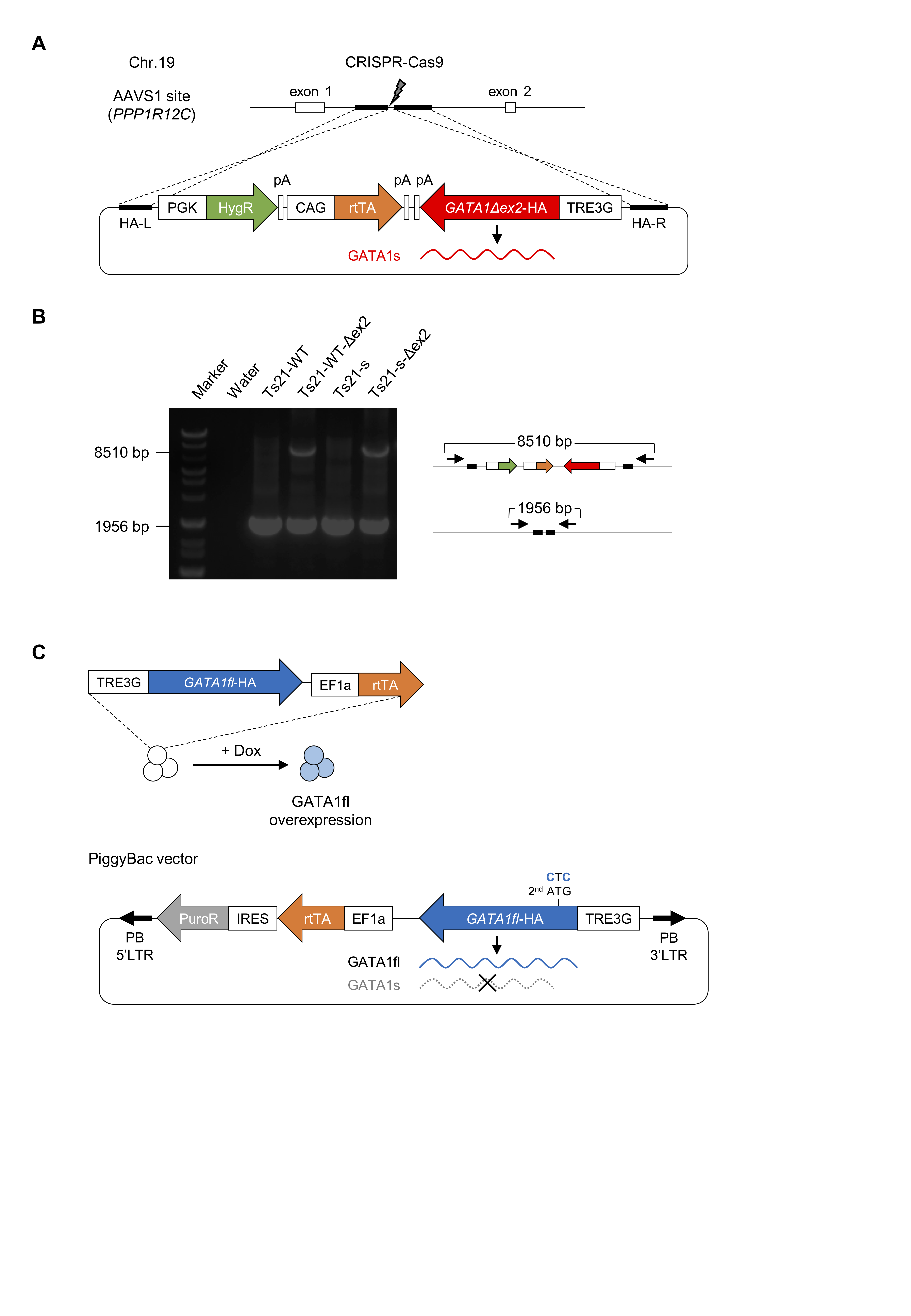

Supplement: S2 Fig — (A) Schematic overview of the AAVS1 targeting strategy by CRISPR-Cas9 to generate Dox-inducible GATA1s for Ts21-ES lines. (B) Genomic PCR to confirm the integration of the Dox-inducible GATA1s cassette. Expected fragment size: integration of Dox-inducible GATA1Δex2-HA, 8510 bp; no integration, 1956 bp. (C) Scheme of Dox-inducible GATA1fl and PiggyBac vector for Dox-inducible GATA1fl. The second ATG was replaced with CTC to express only GATA1fl. (TIFF) [file pone.0247595.s002.tiff]

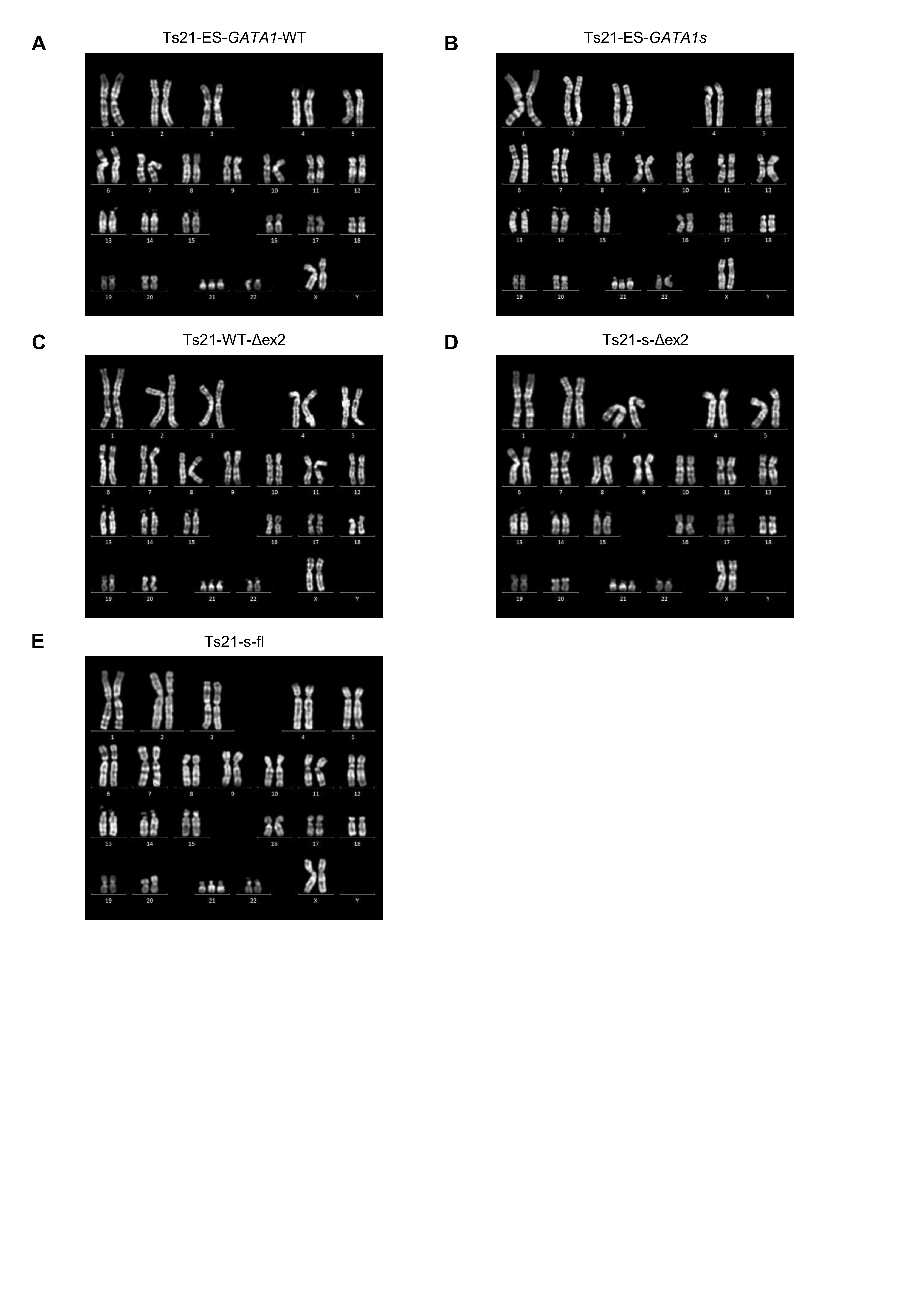

Supplement: S3 Fig — (A-E) Representative Q-banding karyotypes of (A) Ts21-ES-GATA1-WT (Ts21-WT), (B) Ts21-ES-GATA1s (Ts21-s), (C) Ts21-WT-Δex2, (D) Ts21-s-Δex2 and (E) Ts21-s-fl. (TIFF) [file pone.0247595.s003.tiff]

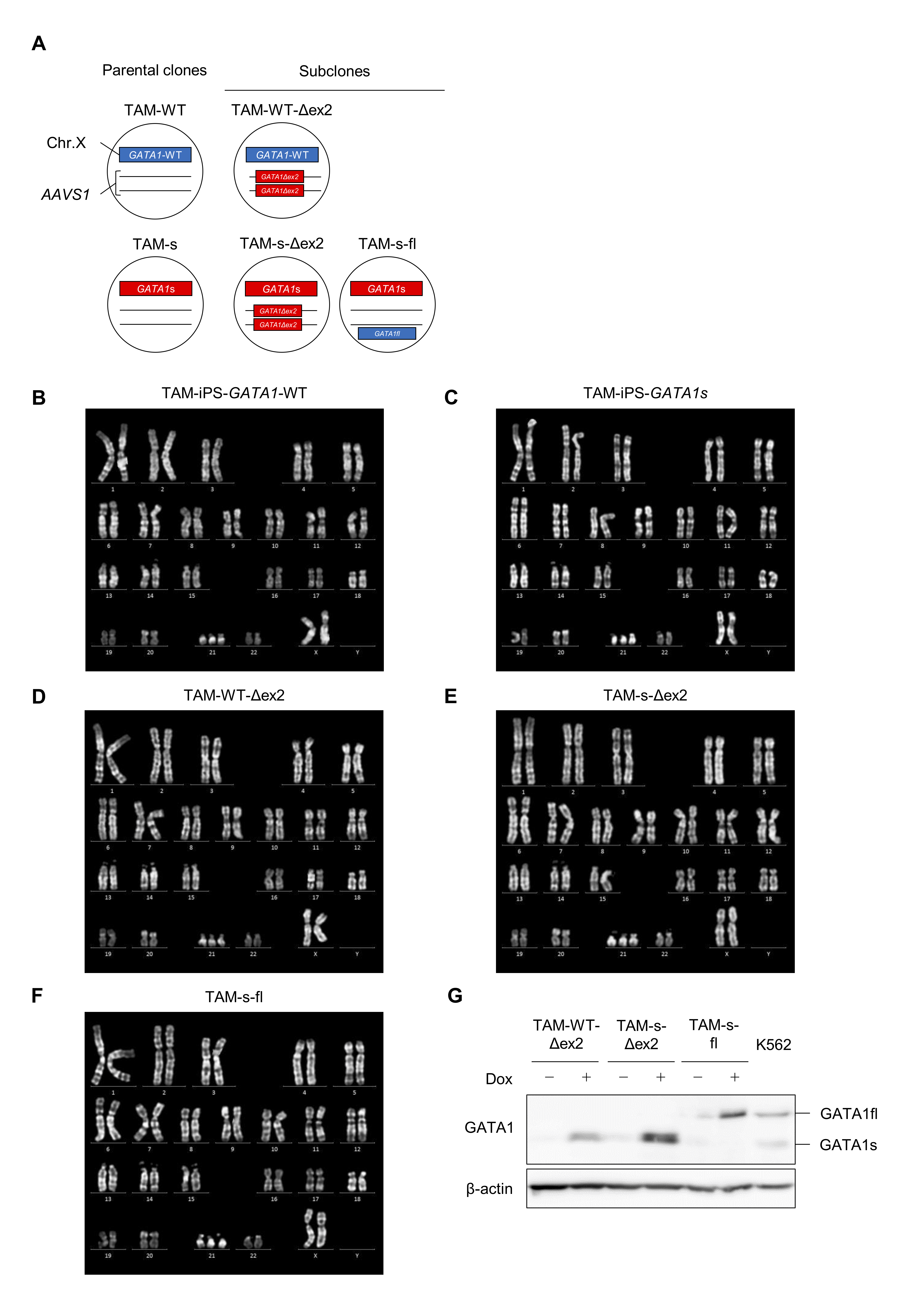

Supplement: S4 Fig — (A) Parental clones and generated GATA1s or GATA1fl Dox-inducible subclones. The Dox-inducible GATAs construct was knocked into AAVS1 locus with CRISPR-Cas9 system, and the Dox-inducible GATA1fl construct was transduced by the PiggyBac system. (B-F) Representative Q-banding karyotypes of (B) TAM-iPS-GATA1-WT (TAM-WT), (C) TAM-iPS-GATA1s (TAM-s), (D) TAM-WT-Δex2, (E) TAM-s-Δex2 and (F) TAM-s-fl. (G) Western blot analysis of GATA1s and GATA1fl expression in untreated iPSCs and iPSCs treated with 1 μg/mL Dox for 24 h. K562 was used as the positive control. (TIFF) [file pone.0247595.s004.tiff]

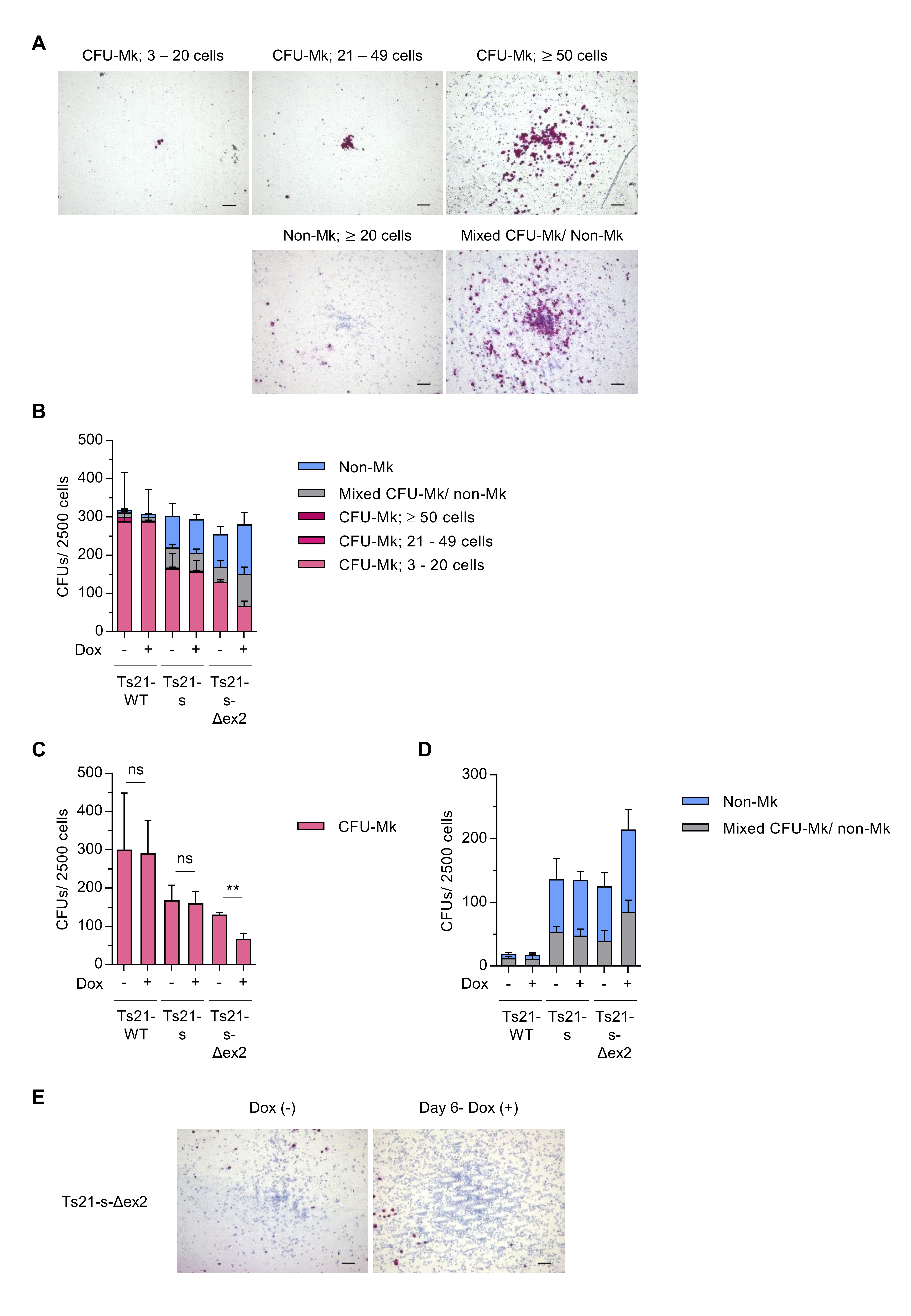

Supplement: S5 Fig — (A) Representative images of each types of colonies in colony-forming unit assay of megakaryocytic progenitors. (B-D) Numbers of CFUs resulting from 2,500 CD235a-CD34+CD43+ cells on day 6 with or without Dox treatment, (B) total, (C) total of CFU-Mk and (D) total of mixed CFU-Mk/ non-Mk and non-Mk (n = 3 biologically independent experiments for Ts21-WT and Ts21-s-Δex2 and n = 4 for Ts21-s). (E) Representative images of non-Mk colonies observed in Dox-untreated and Dox-treated Ts21-s-Δex2. Scale bars: 100 μm. Data are presented as the mean ± SD. **p < 0.01 vs. untreated sample of each clones by two-tailed unpaired Student’s t-test. (TIFF) [file pone.0247595.s005.tiff]

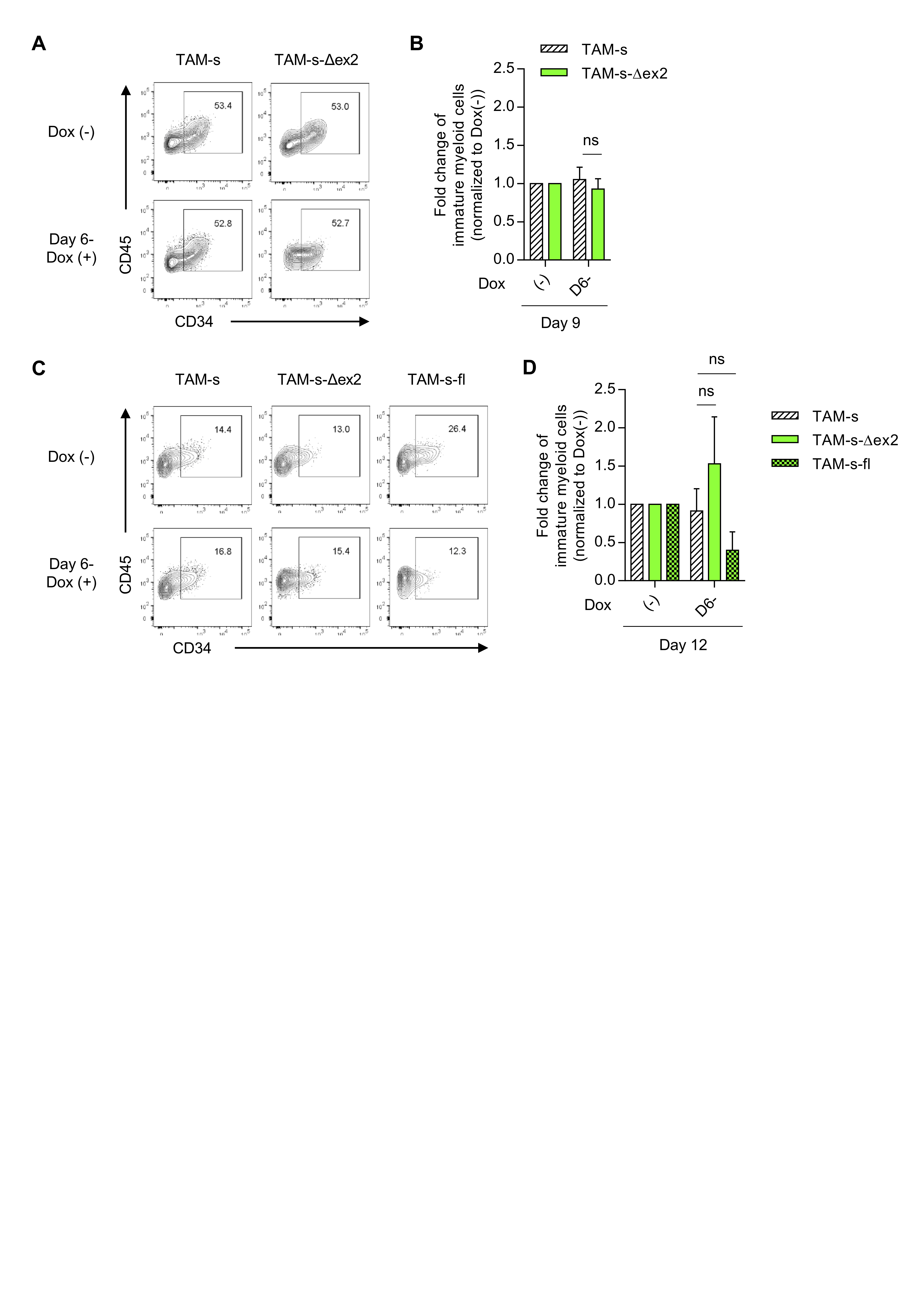

Supplement: S6 Fig — (A) Representative flow cytometry of staining for CD34 and CD45 among myeloid cells on day 9. Upper panels indicate the Dox-untreated sample and lower panels indicate the Dox-treated sample from day 6 for each clone. (B) Fold changes of immature myeloid cells over each untreated sample on day 9. (C) Representative flow cytometry of staining for CD34 and CD45 among myeloid cells on day 12 with or without Dox treatment from day 9. (D) Fold changes of immature myeloid cells over each untreated sample on day 16 (n = 3 biologically independent experiments). Data are presented as the mean ± SD. ns vs. TAM-s under the same treatment by two-tailed unpaired Student’s t-test. (TIFF) [file pone.0247595.s006.tiff]

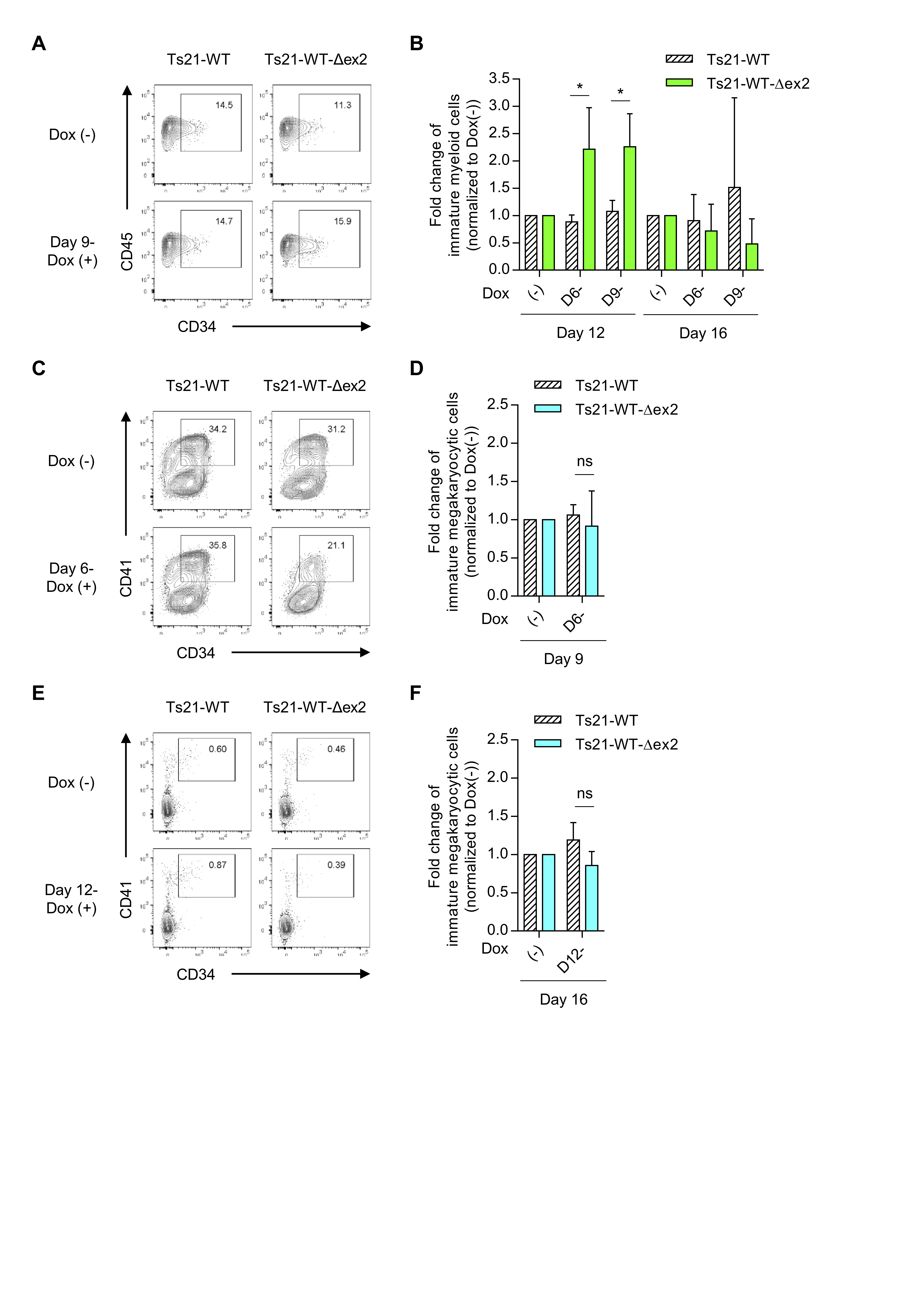

Supplement: S7 Fig — (A) Representative flow cytometry of staining for CD34 and CD45 among myeloid cells on day 12. Upper panels indicate the Dox-untreated sample and lower panels indicate the Dox-treated sample from day 9. (B) The fold changes of immature myeloid cells over each untreated sample on day 12 and day 16. (C, E) Representative flow cytometry of staining for CD34 and CD41 (C) on day 9 with or without Dox treatment from day 6 and (E) on day 16 with or without Dox treatment from day 12. (D, F) The fold changes of immature megakaryocytic cells over each untreated sample (D) on day 9 and (F) on day 16 (n = 4 biologically independent experiments for Ts21-WT and n = 3 for Ts21-WT-Δex2). Data are presented as the mean ± SD. *p < 0.05 vs. Ts21-WT under same treatment by two-tailed unpaired Student’s t-test. (TIFF) [file pone.0247595.s007.tiff]

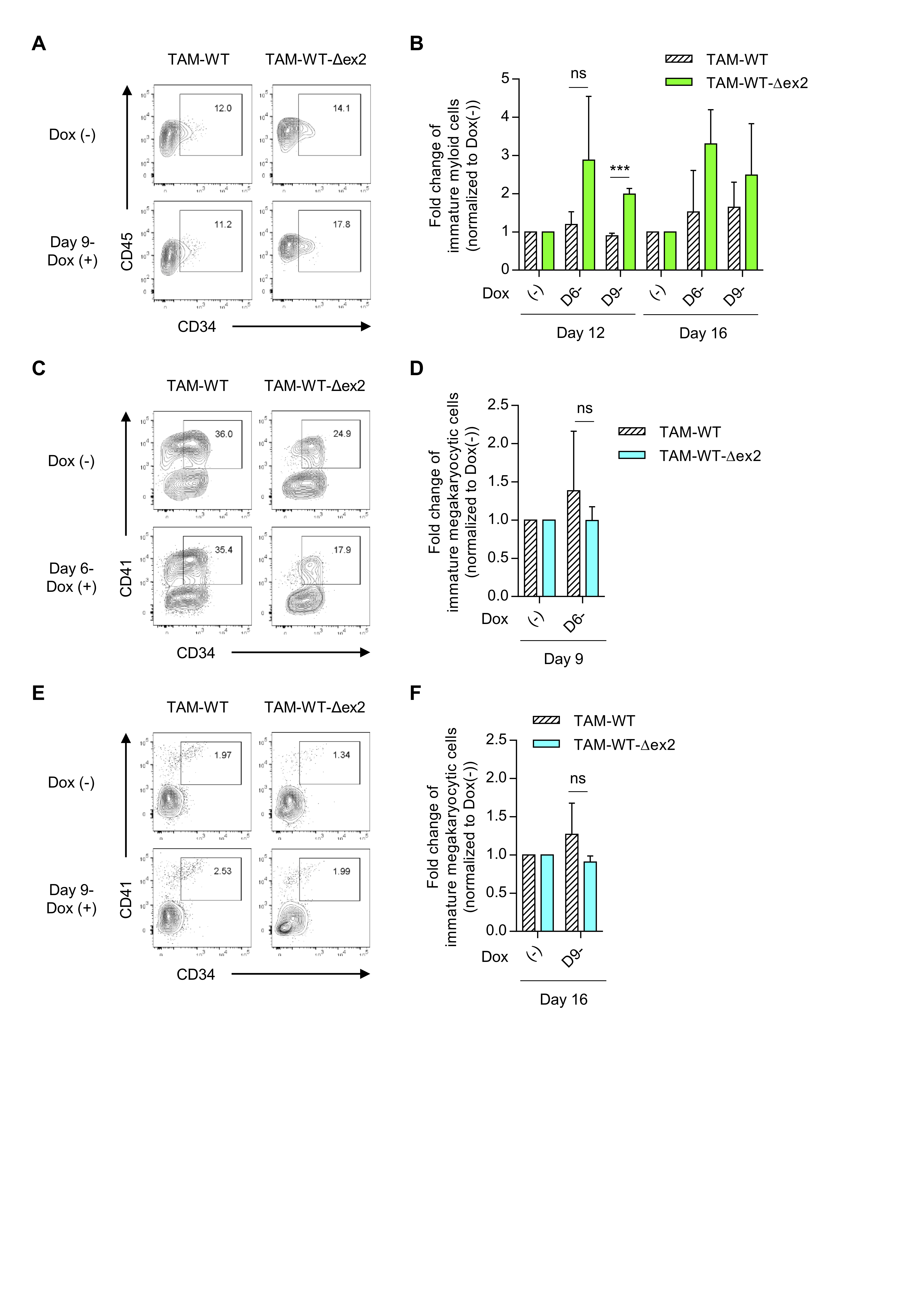

Supplement: S8 Fig — (A) Representative flow cytometry of staining for CD34 and CD45 among myeloid cells on day 12. Upper panels indicate the Dox-untreated sample and lower panels indicate the Dox-treated sample from day 9. (B) The fold change of immature myeloid cells over untreated sample on day 12 and day 16. (C, E) Representative flow cytometry of staining for CD34 and CD41 (C) on day 9 with or without Dox treatment from day 6 and € on day 16 with or without Dox treatment from day 9. (D, F) The fold changes of immature megakaryocytic cells over each untreated sample (D) on day 9 and (F) on day 16 (n = 3 biologically independent experiments). Data are presented as the mean ± SD. ***p < 0.001 vs. Ts21-WT under same treatment by two-tailed unpaired Student’s t-test. (TIFF) [file pone.0247595.s008.tiff]

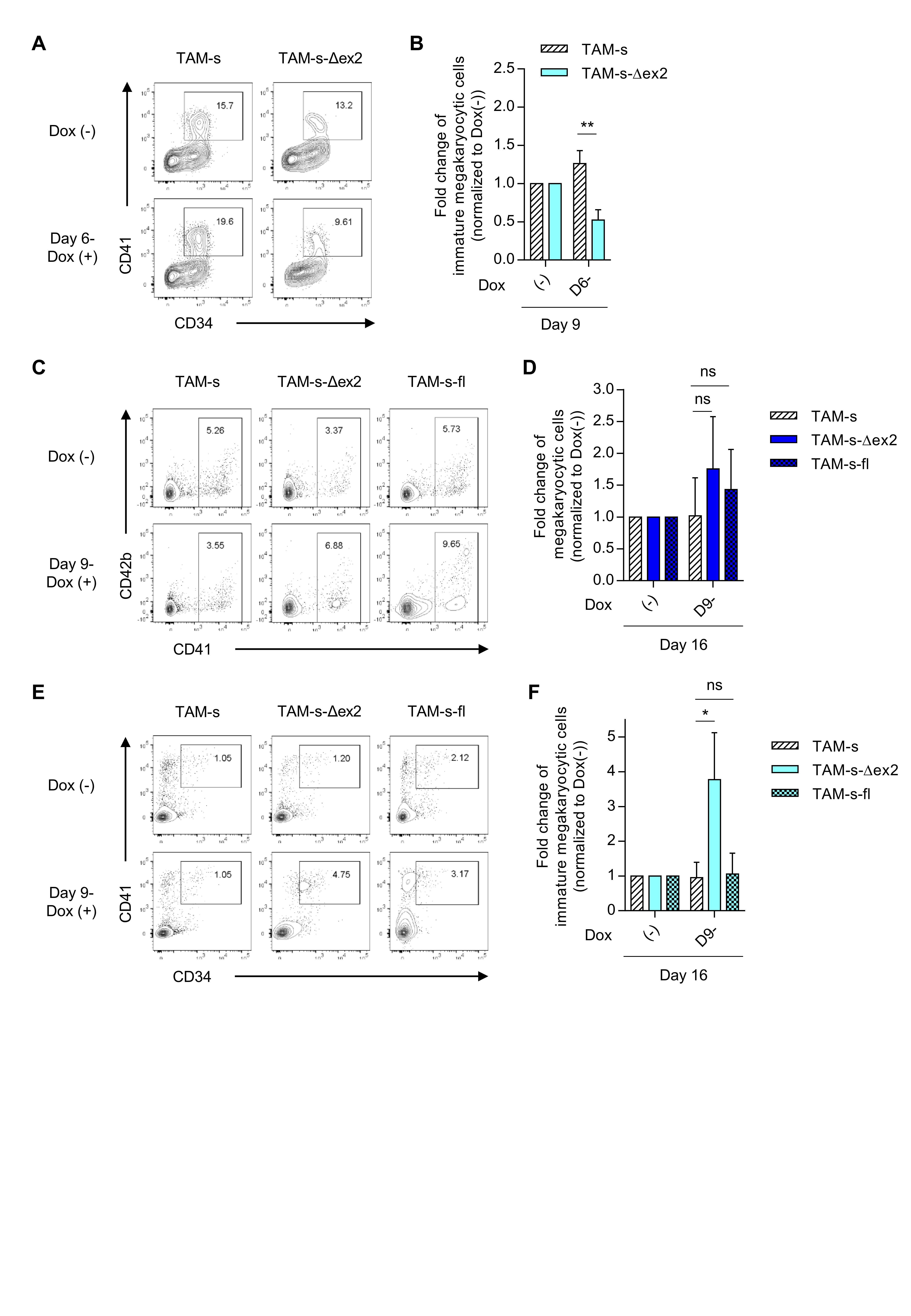

Supplement: S9 Fig — (A) Representative flow cytometry of staining for CD34 and CD41 on day 9. Upper panels indicate the Dox-untreated sample and lower panels indicate the Dox-treated sample from day 6 for each clone. (B) Fold changes of immature megakaryocytic cells over each untreated sample on day 9. (C) Representative flow cytometry of staining for CD41 and CD42b on day 16 with or without Dox treatment from day 9. (D) Fold changes of megakaryocytic cells over each untreated sample on day 16. (E) Representative flow cytometry of staining for CD34 and CD41 on day 16 with or without Dox treatment from day 9. (F) Fold changes of immature megakaryocytic cells over each untreated sample on day 16 (n = 3 biologically independent experiments). Data are presented as the mean ± SD. *p < 0.05, **p < 0.01 by two-tailed unpaired Student’s t-test. (TIFF) [file pone.0247595.s009.tiff]

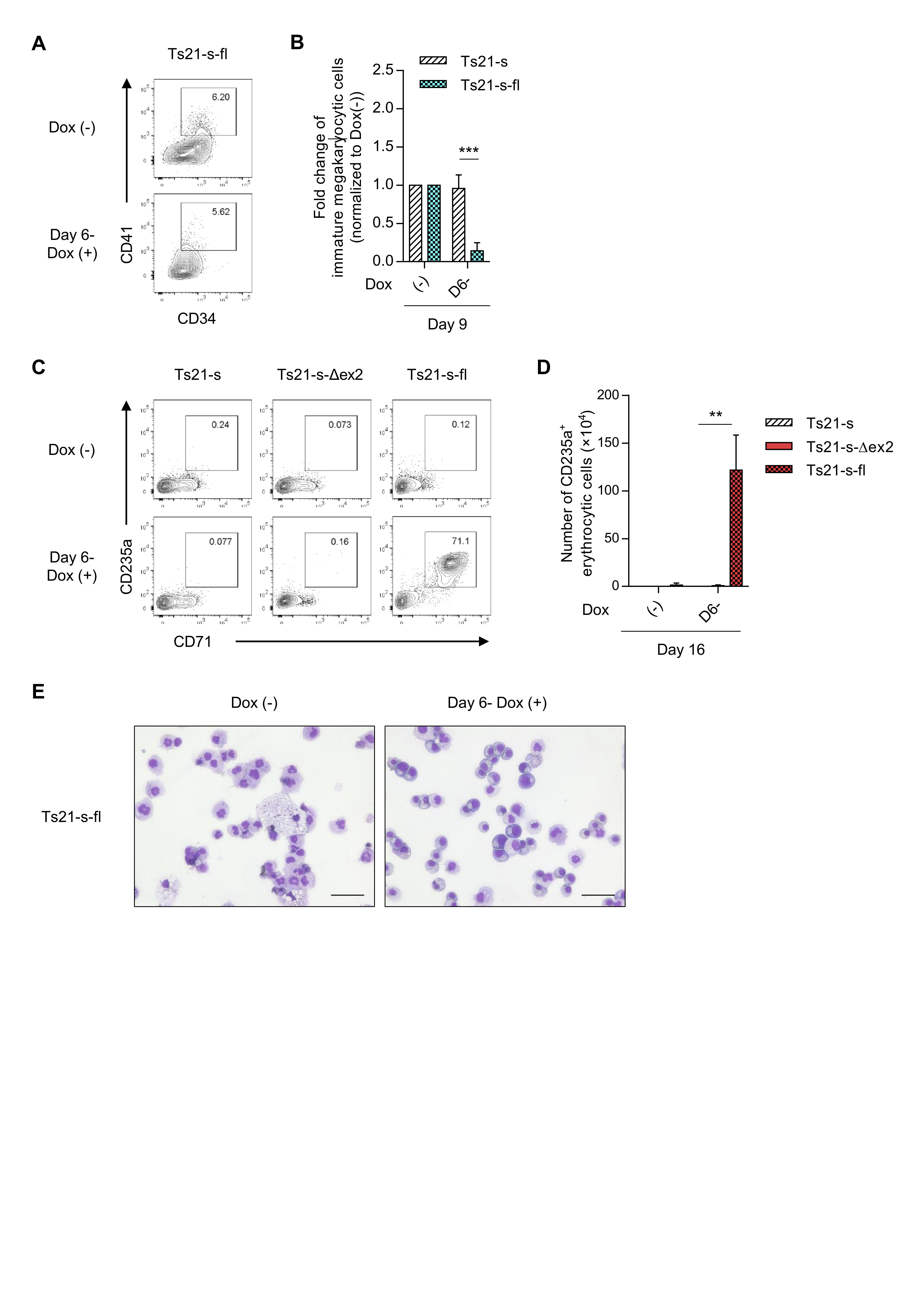

Supplement: S10 Fig — (A) Representative flow cytometry of staining for CD34 and CD41 on day 9. Upper panels indicate the Dox-untreated sample and lower panels indicate the Dox-treated sample from day 6. (B) The fold changes of immature megakaryocytic cells over each untreated sample on day 9. (C) Representative flow cytometry of staining for CD71 and CD235a on day 16 with or without Dox treatment from day 6. (D) Average number of CD235a+ erythrocytic cells on day 16 (n = 5 biologically independent experiments for Ts21-s and n = 3 for Ts21-s-fl). (E) May-Giemsa staining of Ts21-s-fl on day 16 with or without Dox treatment from day 6. Scale bars: 50 μm. Data are presented as the mean ± SD. **p < 0.01, ***p < 0.001 vs. Ts21-s under same treatment by two-tailed unpaired Student’s t-test. (TIFF) [file pone.0247595.s010.tiff]

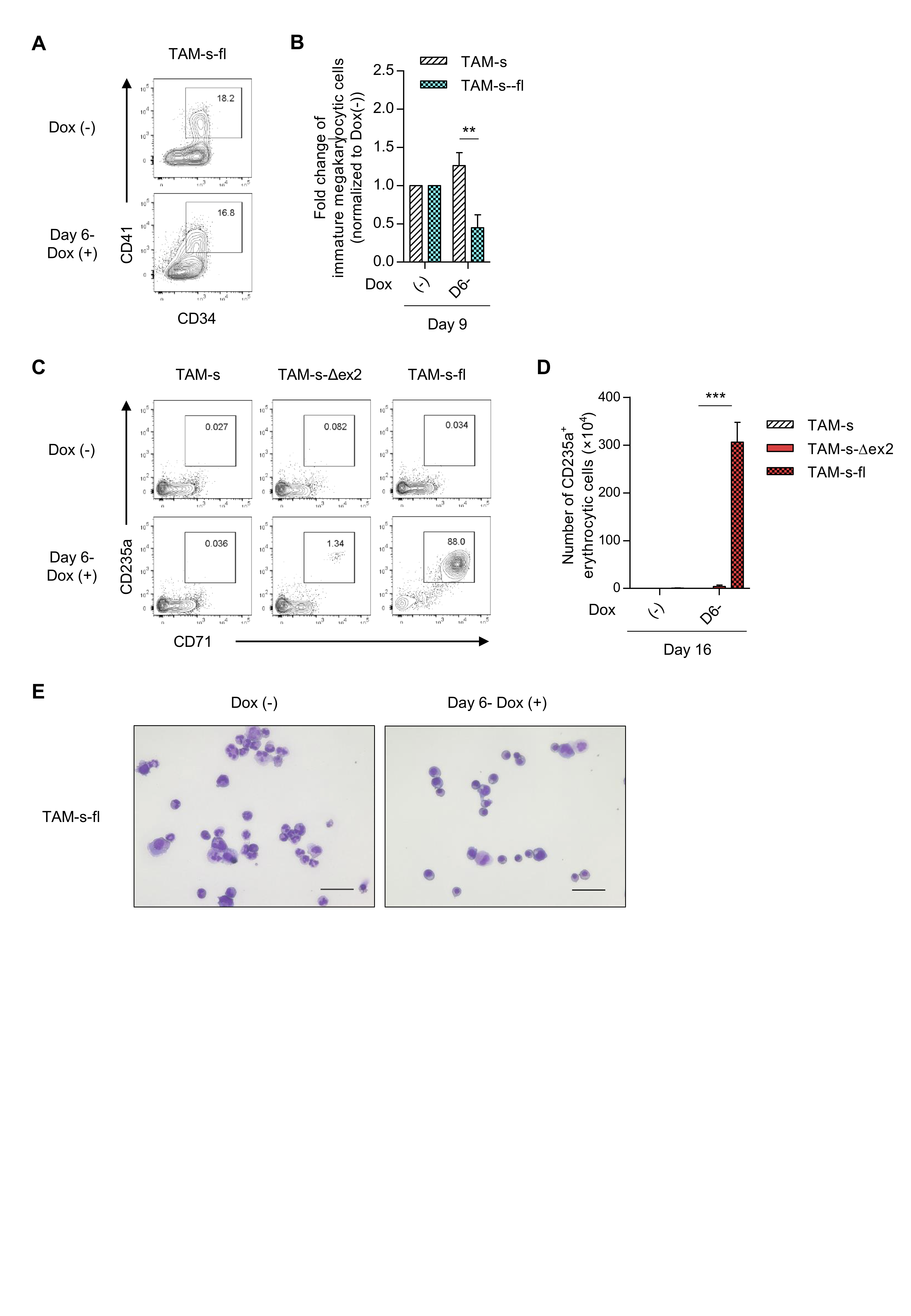

Supplement: S11 Fig — (A) Representative flow cytometry of staining for CD34 and CD41 on day 9. Upper panels indicate the Dox-untreated sample and lower panels indicate the Dox-treated sample from day 6. (B) The fold changes of immature megakaryocytic cells over each untreated sample on day 9. (C) Representative flow cytometry of staining for CD71 and CD235a on day 16 with or without Dox treatment from day 6. (D) Average number of CD235a+ erythrocytic cells on day 16 (n = 3 biologically independent experiments). (E) May-Giemsa staining of TAM-s-fl on day 16 with or without Dox treatment from day 6. Scale bars: 50 μm. Data are presented as the mean ± SD. **p < 0.01, ***p < 0.001 vs. TAM-s under same treatment by two-tailed unpaired Student’s t-test. (TIFF) [file pone.0247595.s011.tiff]

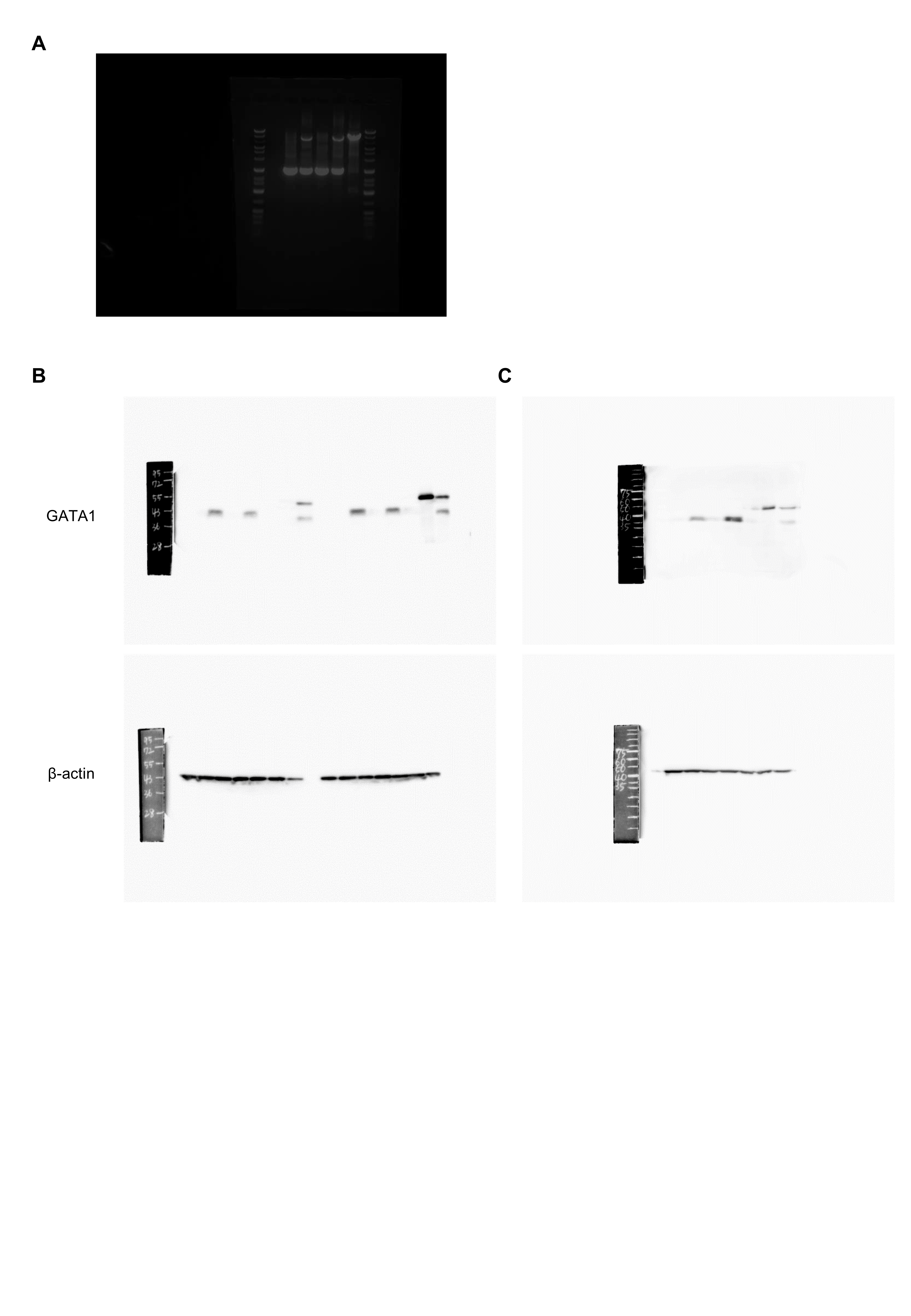

Supplement: S12 Fig — (A) The original image of electrophoretic gel of S2B Fig. lane 1, Marker; lane 2, water; lane 3, Ts21-WT; lane 4, Ts21-WT-Δex2; lane 5, Ts21-s; lane 6, Ts21-s-Δex2; lane 7–8, not shown. (B, C) Original uncut gel images of western blot analysis. (B) Ts21-ES clones on Fig 2C; lane 1–7, not shown; lane 8, Marker; lane 9, Ts21-WT-Δex2 Dox (-); lane 10, Ts21-WT-Δex2 Dox (+); lane 11, Ts21-s-Δex2 Dox (-); lane 12, Ts21-s-Δex2 Dox (+); lane 13, Ts21-s-fl Dox (-); lane 14, Ts21-s-fl Dox (+); lane 15, K562. (B) TAM-iPS clones on S4G Fig; lane 1, Marker; lane 2, TAM-WT-Δex2 Dox (-); lane 3, TAM-WT-Δex2 Dox (+); lane 4, TAM-s-Δex2 Dox (-); lane 5, TAM-s-Δex2 Dox (+); lane 6, TAM-s-fl Dox (-); lane 7, TAM-s-fl Dox (+); lane 8, K562. (TIFF) [file pone.0247595.s012.tiff]
