# Supplementary material for: Pluripotent stem cell model of early hematopoiesis in Down syndrome reveals quantitative effects of short-form GATA1 protein on lineage specification
Source: PLoS One. 2021 Mar 29;16(3):e0247595. doi: 10.1371/journal.pone.0247595 (PMC8007000; doi:10.1371/journal.pone.0247595)
Supplement: S1 File — (DOCX) [file pone.0247595.s013.docx]

**Supplemental materials and methods**

**Karyotyping**

For karyotyping, cultured PSCs were incubated with 0.4 μg/mL KaryoMAX^TM^ Colcemid^TM^ solution (Thermo Fisher Scientific) for 1 h. Dissociated cells were treated with hypotonic solution and fixed with carnoy solution. Karyotyping of fixed samples was requested to Trans Chromosomics Co., Ltd. (Tottori, Japan).

**Colony-forming unit assay of megakaryocytic progenitors**

Colony-forming unit assay of megakaryocytic progenitors (CFU-Mk) was performed with MegaCult^TM^-C with cytokines (#04901, STEMCELL technologies, Vancouver, Canada). 2.5×10^3^ sorted CD235a^-^CD34^+^CD43^+^ cells on day 6 were plated in MegaCult^TM^-C collagen based medium with or without 1 µg/mL Dox and cultured for 10 days. Staining for GPⅡb/Ⅲa antibody and scoring of CFU colonies were performed in accordance with the manufacturer’s instructions.
